# Glutamine potentiates cefoperazone-sulbactam activity against *Pseudomonas aeruginosa* by increasing membrane permeability and cellular uptake

**DOI:** 10.3389/fmicb.2025.1631646

**Published:** 2025-07-03

**Authors:** Jiao Xiang, Xin Wang, Huiyin Lin, Lifen Yang, Xiaoxia Huang, Yuetao Chen, Yingyue Zeng, Shaohua Li, Xianliang Zhao, Shiwen Wang, Yuan Tao, Huanzhe Fu, Zhengqi Shi, Kuihai Wu, Xuanxian Peng, Hui Li, Jin Tang, Zhuanggui Chen

**Affiliations:** ^1^State Key Laboratory of Bio-Control, Third Affiliated Hospital, School of Life Sciences, Sun Yat-sen University, Guangzhou, China; ^2^Hanzhong Central Hospital, Hanzhong, China; ^3^Guangdong Provincial Key Laboratory of Marine Biotechnology, Department of Biology, Shantou University, Shantou, China; ^4^Department of Laboratory Medicine, The First People’s Hospital of Foshan, Foshan, China

**Keywords:** preclinical pharmacodynamics, cefoperazone/sulbactam, multidrug resistance, carbapenem resistance, glutamine, *Pseudomonas aeruginosa*

## Abstract

**Purposes:**

The combination of an antibiotic with a metabolic reprogramming agent is anticipated to emerge as a promising therapeutic strategy against antibiotic-resistant bacteria, though this hypothesis requires validation through preclinical pharmacodynamic studies.

**Methods:**

This study evaluated the preclinical pharmacodynamic profile of cefoperazone-sulbactam (SCF) combined with glutamine against *Pseudomonas aeruginosa* clinical isolates, including 54 antibiotic-sensitive (S-PA), 20 multidrug-resistant (MDR-PA), and 185 carbapenem-resistant strains (CR-PA).

**Results:**

The combination demonstrated synergistic efficacy in 251 cases (96.9%), equivalence in 7 (2.7%), and no interaction in 1 (0.4%) compared to SCF monotherapy. Time-kill assays, bacterial load quantification, and murine infection models consistently validated these findings, with therapeutic effects remaining stable by calcium concentrations and pH gradients. Glutamine slows the development of SCF resistance, delays the post-antibiotic effect, and reduces mutation frequency. Mechanistically, glutamine reprograms bacterial metabolism from an antibiotic-resistant to an antibiotic-sensitive state, thereby enhancing membrane permeability and increasing drug uptake. This accelerated drug influx surpasses the clearance capacity mediated by efflux pumps and enzymatic degradation, resulting in increased bacterial eradication.

**Conclusion:**

These findings suggest that the synergistic combination holds potential for developing therapeutic candidates against MDR-PA and CR-PA.

## 1 Introduction

*Pseudomonas aeruginosa* is a versatile opportunistic pathogen. This organism employs sophisticated intrinsic and acquired resistance mechanisms to counteract most conventional antibiotics. Clinically, *P. aeruginosa* is implicated in chronic progressive pulmonary infections and persists as a leading cause of life-threatening conditions with substantial morbidity and mortality (Wood et al., 2023). The emergence of multidrug-resistant *P. aeruginosa* (MDR-PA), particularly carbapenem-resistant strains (CR-PA), correlates with alarmingly elevated mortality rates (Al-Orphaly et al., 2021; Zhang et al., 2022). In recognition of this threat, the World Health Organization (WHO) has classified CR-PA as a critical-priority pathogen necessitating the urgent development of targeted therapeutics (Tacconelli et al., 2018; Soukarieh et al., 2024). These pressing challenges underscore the critical need for innovative antimicrobial strategies to combat MDR-PA and CR-PA infections. Indeed, novel therapeutic strategies are being developed (Yin et al., 2024), including the identification of new antibacterial targets (Li and Ma, 2015).

Antibiotic resistance mechanisms are broadly categorized into four types: reduced membrane permeability, enhanced enzymatic degradation, increased efflux activity, and target modification (Peng et al., 2019). These mechanisms are further classified into two functional groups: (1) prevention of extracellular antibacterial agent entry (PEEAA) through diminished membrane permeability, and (2) dampening of intracellular antibacterial agent activity (DAIAA) via the remaining three mechanisms (Peng et al., 2023). DAIAA operates within the drug concentration threshold permitted by PEEAA, implying that sufficient drug influx can override DAIAA-mediated resistance mechanisms, including β-lactamase activity. *P. aeruginosa* develops carbapenem insensitivity through dual strategies: production of carbapenemases and impairment of membrane permeability (Tenover et al., 2022; Yoon and Jeong, 2021). Carbapenemases, the most versatile class of β-lactamases, exhibit broad-spectrum hydrolytic activity against nearly all β-lactam antibiotics, including carbapenems (Cherak et al., 2021; García-Betancur et al., 2021). Additionally, the bacterium has high tendency for biofilm formation, which protects the cells from antibiotics (Thi et al., 2020). Concurrently, reduced membrane permeability and biofilm restricts intracellular accumulation of carbapenems and other antimicrobials, causing sublethal drug concentrations. Thus, enhancing membrane permeability to maximize drug influx represents a viable strategy to counteract antibiotic-resistant *P. aeruginosa*.

Emerging metabolic state reprogramming has emerged as a strategic approach to combat antibiotic resistance by reverting drug-resistant bacteria from an antibiotic-resistant metabolic profile to a drug-sensitive state, thereby potentiating antimicrobial uptake (Peng et al., 2015a, 2015b). This paradigm enables the revitalization of existing antibiotics against resistant pathogens through mechanism-based synergy (Peng et al., 2015b; Xiang et al., 2024; Peng et al., 2024, 2025). Our prior work demonstrated that exogenous glutamine enhances ampicillin accumulation in multidrug-resistant *Escherichia coli* via the glutamine-inosine-CpxA/R-OmpF regulatory axis, achieving intracellular concentrations surpassing intrinsic resistance thresholds. This enhancement is also observed when combined with other clinically commonly used antibiotics, including cephalosporins (Zhao et al., 2021). In this study, we systematically evaluated the combination of glutamine with cefoperazone-sulbactam (SCF) against drug-resistant *P. aeruginosa*, adhering to standardized preclinical pharmacodynamic protocols for novel antibacterial agents (Li et al., 2023; Hong and Qin, 2023). SCF was selected because it is a broad-spectrum antibiotic and commonly used in clinical practice to treat *P. aeruginosa* infections (Lai et al., 2023; Ku and Yu, 2021). The resultant pharmacodynamic profile confirms the therapeutic efficacy of this combination against MDR-PA and CR-PA strains.

## 2 Materials and methods

### 2.1 Main reagents and their preparing

Main reagents involved in this paper included SCF (Qilu Antibiotics Co., Ltd., China), glutamine (Guangzhou HC Pharmaceutical Co., Ltd., China), calcium chloride, M9 medium, LB (Luria-Bertani) medium. SCF was dissolved in sterilized double distilled H_2_O (ddH_2_O), subpacked in 1 mL tube, kept in −80°C. When it was used, the subpacked SCF was diluted into desired concentration by one or two times and used once. Glutamine was aseptically weighed 292.28, freshly dissolved in 200 mL of M9 medium, and diluted into the desired concentration for the same day use only. Calcium chloride with 1 M was prepared as stock solution and diluted into working concentration using M9 medium. LB medium was prepared in 1 L deionized water with 10 g bacteriological peptone, 10 g sodium chloride, 5 g yeast extract powder and pH value of 7.0–7.4 adjusted by 5 M sodium hydroxide. M9 medium was prepared in 1 L deionized water with 17.1 g Na_2_HPO_4_-12H_2_O (sigma), 3 g KH_2_PO_4_ (sigma), 1 g NH_4_Cl (sigma), 0.5 g NaCl (sigma), 20 mM lysine. Mueller–Hinton Broth (MHB) medium was prepared by weighing 22.0 g Mueller–Hinton solid powder and dissolving it in 1 L distilled water.

### 2.2 Bacterial isolates and MIC testing

*P. aeruginosa* isolates were collected between 2021 and 2023 from Xiamen University Affiliated Zhongshan Hospital (Fujian, China); the First People’s Hospital of Foshan (Guangdong, China); Hanzhong Central Hospital (Shanxi, China); and the Third Affiliated Hospital of Sun Yat-sen University (Guangdong, China). All isolates were cultured in LB medium at 37°C with shaking at 200 rpm for 18 h. MIC is defined as the lowest concentration of an antimicrobial agent required to inhibit the visible growth of a microorganism after a specified incubation period. The MIC values were determined using the microdilution broth susceptibility assay as recommended by the Clinical & Laboratory Standards Institute (CLSI, 2023). Serial twofold dilutions of antibiotic (from 120 to 0.05 μg/mL) were prepared in a 96-well plate (100 μL per well). Wells with no antibiotic were used as positive growth control. A diluted bacterial suspension in LB medium was added to each well to give a final concentration of 5 × 10^4^ colony-forming units (CFU)/mL, confirmed by viable counts. Wells without bacteria were used as a negative growth control. The plate was incubated for 16 h at 37°C and growth was visually assessed. At least three independent determinations were done.

### 2.3 Antibiotic bactericidal assays

Antibiotic bactericidal assays were conducted as described previously (Allison et al., 2011). Three or four bacterial colonies were cultured in test tubes containing 5 mL LB medium at 37°C for 16 h. After centrifugation at 8,000 rpm for 5 min, samples were washed twice with sterile saline and resuspended in M9 minimal medium containing 20 mM lysine as a carbon source. The bacterial solution was adjusted to an OD_600_ of 0.2, diluted 300-fold, and used for subsequent experiments. Desired concentrations of SCF (3 μg/mL for S-PA, 50 μg/mL for MDR-PA, and 50 μg/mL for CR-PA and 20 mM glutamine were added), and samples were incubated at 37°C for 6 h. Cultures were serially diluted, plated on LB agar, and incubated at 37°C for 16–22 h. Colony-forming units (CFUs) were enumerated for dilutions yielding 20–200 colonies, and percent survival was calculated relative to untreated controls. In addition to evaluating individual bactericidal activity using percent survival, we established population-level killing efficiency metrics: killing efficiency 50 (KE50) and killing efficiency 90 (KE90). The terms KE50 and KE90 represent the killing efficiency (expressed as a percentage) required to eliminate the 50th and 90th percentile of bacterial strains in a tested population, respectively. This metric was established because a value analogous to MIC50 and MIC90 was needed to evaluate the bactericidal efficacy of methods across a bacterial population.

### 2.4 Phenotypic characterization of carbapenemase production

Screening of carbapenemase phenotypes and identification of metallo-β-lactamases (MBLs) were performed according to the modified carbapenem inactivation method/ethylenediaminetetraacetic acid-modified carbapenem inactivation method (mCIM/eCIM) recommended by the USA CLSI guidelines (CLSI, 2023). Saturated bacteria from overnight incubation at 37°C were inoculated into two 3 mL tryptic soy broth (TSB) medium (1:2,000) tubes and incubated at 37°C and 200 rpm for 4 h for mCIM and eCIM assays, respectively (30 μL of 0.5 mol/L ethylenediamine tetraacetic acid (EDTA) was added to this tube). *E. coli* ATCC25922 was used as the control strain for the enzyme yield assay, cultured overnight to saturation, diluted with saline to OD_625_ = 0.08–0.13, and 100 μL was coated on MHB plates. After the plates were dried, the drug-sensitive paper pieces were taken out from two tubes of TSB broth of the strains to be tested and pasted on the same MHB plate, making sure that the distance between the center of the paper pieces was not less than 24 mm, and the distance between the paper pieces and the edges was not less than 15 mm. When the diameter of the inhibition circle formed by the 10 μg meropenem paper pieces was greater than or equal to 19 mm, then there was not carbapenem-resistant *Enterobacteriaceae*; if the diameter of the inhibition circle was less than 19 mm but greater than 15 mm, or the inhibition circle had scattered colonies, then it was judged to be intermediate. If it is less than 19 mm but greater than 15 mm or there are sporadic colonies in the inhibition circle, it is judged to be intermediate; if it is less than or equal to 15 mm, it is judged to be producing carbapenemases; when it is judged to be producing carbapenemases, it can be further judged to see if it is producing metalloenzymes or otherwise. This judgment method is compared with the use of meropenem alone, meropenem paper combined with EDTA, the diameter of the circle of inhibition is greater than or equal to 5 mm, it is judged to produce MBL.

### 2.5 Phenotypic characterization of ESBL and AmpC production

These enzymes were measured according to the methods as recommended by CLSI (2023). The bacterial solution was diluted as described above and 100 μL was taken and spread it evenly on the MHB plate. After drying, 30 μg cefotaxime (CTX) and 30 μg CTX −10 μg clavulanic acid (CTL) composite paper sheets were pasted on the same MHB plate (Oxoid Company, United Kingdom), and 30 μg ceftazidime (CAZ) and 30 μg CAZ with 10 μg clavulanic acid (CAL) (Oxoid Company, United Kingdom). The plates were incubated in an inverted incubator at 37°C for 16 h. The diameter of the circle of inhibition was measured and photographed. When the bacteria produce ESBLs, due to the inhibiting effect of the inhibitor on ESBLs, the circle of inhibition formed by the composite paper sheet of CAL or CTL will be larger than that formed by the separate paper sheet of CAZ or CTX. If the diameter of the circle of inhibition formed by the use of the composite of CCV or CTC is larger than or equal to the diameter of the circle of inhibition formed by the corresponding single paper sheet, it will be judged as a positive ESBLs-producing bacterial strain. AmpC β-lactamase (AmpC‌) were detected by the K-B paper diffusion method. The experimental procedure was the same as the ESBLs assay, and the strain was suspected of producing AmpC when the diameter of the circle of inhibition of the 30 μg cefoxitin (FOX)-resistant paper sheet was less than or equal to 18 mm.

### 2.6 Quantification of intracellular cefoperazone

Quantification of intracellular cefoperazone was described previously (Zhang et al., 2022; Monteiro et al., 2018). MDR-PA 0652 was cultured in 16 h and adjusted the bacterial solution OD_600_ to 0.2. The solution was divided into black control group, SCF group, glutamine group, and SCF + glutamine group. The experiments were involved in SCF concentration (75, 125, 250, 500, and 1,000 μg/mL) with and without glutamine concentration (0, 0.01, 0.1, 1, 5, and 20 mM) and incubation time (0, 1, 2, 3, 4, 5, and 6 h). These mixtures were mixed and incubated at 37°C and 200 rpm for 6 h. These cultures were collected and washed with saline for 3 times and then adjusted into 1.0 of OD_600_. Aliquots of 10 mL were centrifuged for the collection of bacterial cells. The cells were resuspended in 1.0 mL of phosphate buffer saline (PBS) and then subjected to ultrasonic crushing (35% of the power, 2 s of operation, and a pause of 3 s). The ultrasonically broken solution was centrifuged at 12,000 rpm for 10 min and the supernatant was used for liquid chromatography-mass spectrometry (LC-MS) analysis and bacterium-killed test. For LC-MS analysis, the chromatographic column was a Hypersil GOLDTM HPLC C18 (100 × 2.1 mm, Partical Sz. 1.9 μm) column from Thermoscientific, and the liquid phase separation was performed by gradient elution. The injection volume was 2 μL and the column temperature was 40°C. Mobile phase A: 0.1% (v/v) formic acid in water and mobile phase B: 100% acetonitrile. A parent ion full scan (Mass Scan/Parent Scan) was performed on a standard working solution of 1 μg/mL of cefoperazone in electrospray negative ionization mode ESI (−) to determine that the protonated molecular ion [M–H]^−^ of cefoperazone was 644 (M is the molecular weight of cefoperazone). After determining the parent ion of cefoperazone, secondary mass spectrometry of the molecule was carried out using the Daughter Scan method (Daughter Scan), and by adjusting the collision voltage, the three with high abundance and non-interference with each other were selected as the daughter ions: 655 > 140, 655 > 188, and 655 > 214, of which the highest abundance was used as the quantitative ion, and 655 > 140 as the qualitative ion. In this experiment, the external standard method was used and the measured results were calculated using Xcalibur software. For bacterium-killed, 50 μL of supernatant was transferred to a 1.5 mL Eppendorf tube containing 450 μL of M9 medium supplemented with K12 strain, which was prepared by 1:100 dilution of overnight saturated culture to an OD_600 nm_ value of 1.0, followed by 200,000-fold dilution. After incubation at 37°C with 200 rpm shaking for 6 h, bacterial growth was assessed through CFU/mL. Cefoperazone concentrations were determined using a standard curve. For standard curve construction, Eppendorf tubes were prepared with cefoperazone concentrations of 4,000, 2,000, 1,000, 500, 250, and 0 ng, respectively.

### 2.7 Pharmacokinetics of SCF in a CR-PA mode

This assay was performed as previously described (Kojima and Nikaido, 2013; Zhao et al., 2021). The effect of glutamine addition on the osmotic rate of SCF entry into bacteria (*V*_in_) and the efflux rate of efflux from the bacterial efflux pump (*V*_e_) was determined. *V*_e_ = *V*_emax_ × (*C*_p_)*h*/[(*K*_0.5_)*h* + (*C*_p_)*h*], where *V*_emax_ (maximal efflux rate of 0.37 nmol/mg/s), *K*_0.5_ (half-maximal efflux concentration of the drug 0.94 μM), and *h* (Hill’s coefficient 0.23). *C*_p_ was calculated as the intracellular cefoperazone content determined above. Without the addition of glutamine, *V*_in_ was calculated as *V*_in_ = *P* × *A* × (*C*_0_ − *C*_p_). The *C*_0_ value is the 1× MIC value of 32 μg/mL (equivalent to 0.0387 mM) for a SCF concentration. With the addition of glutamine, *V*_in_ is calculated as *V*_in_ = *P* × *A* × (*C*_p_ without glutamine/*C*_p_ with glutamine) × (*C*_0_ − *C*_p_). In the above equation, *P* is the osmotic coefficient (0.28 × 10^−5^ cm/s) and *A* is the cell surface area (constant value, 10^3^ cm^2^/mg).

### 2.8 Sample preparation for GC-MS analysis

Samples preparation for the metabolomes was carried out according to the previously described (Peng et al., 2015b). Briefly, bacterial cultures were incubated at 37°C with 200 rpm for 16 h in the LB medium. Subsequently, the cultures were inoculated into 50 mL of LB medium at a ratio of 1:100, and cultured at 200 rpm until the optical density (OD_600 nm_) reached 1.0. Then, the bacteria were harvested and washed two times with sterile saline solution. The cells were resuspended in saline and adjusted to an optical density (OD_600 nm_) of 1.0. Each 10 mL aliquot of the cells was collected and transferred to a 1.5 mL centrifuge tube. The cells were immediately quenched with −80°C pre-cooled methanol (HPLC grade). The quenched cells were then sonicated for 10 min (650 W total power with a 35% output, 2 s pulse, 3 s pause) on ice, with the addition of 10 μL of 0.1 mg/mL ribitol (Sigma) as an internal standard. The resulting mixture was centrifuged at 4°C and 12,000 × g for 10 min to obtain the supernatant, which was transferred to a 37°C vacuum centrifuge dryer (Labconco, United States) for methanol evaporation. The dried samples were treated with 80 μL of 20 mg/mL methoximation-pyridine hydrochloride (Sigma-Aldrich) at 37°C and 200 rpm for 3 h. Subsequently, 80 μL of N-methyl-N-trimethylsilyltrifluoroacetamide (MSTFA, Sigma-Aldrich) was added, and the reaction was carried out at 37°C and 200 rpm for 30 min.

### 2.9 Metabolomic data analysis

Identification of metabolites corresponding to the chromatographic peaks in the GC-MS analysis was performed using the NIST Mass Spectral Library. The data were normalized by applying total amount correction and generating standardized datasets consisting of metabolite information, retention times, and peak areas, which were further processed for metabolomics analysis. Significant differences in the standardized data were calculated and selected using IBM SPSS Statistics 19 software, considering a threshold of *p*-value <0.01. Cluster analysis was performed using R software (R×64 3.6.1). The normalized areas of differential metabolites were analyzed using *Z*-score. Principal component analysis and S-plot analysis were carried out using SIMCA-P+12.0 software (version 12; Umetrics, Umea, Sweden), while metabolic pathway analysis was performed using MetaboAnalyst 4.0 enrichment. Figures were created using GraphPad Prism 8.0 and Adobe Illustrator CS6.

### 2.10 Effect of calcium and pH on killing efficacy

The effect of calcium and pH on killing efficacy was carried out as described previously (Schwalbe et al., 2007; Wu et al., 2009). Nineteen isolates (15 CR-PA and 4 MDR-PA) and ATCC27853 were tested for calcium- and pH-dependent killing efficacy. Three or four clones of these bacterial strains were selected and inoculated into test tubes containing 5 mL of LB and incubated at 37°C and 200 rpm for 16 h. These cultures were centrifuged at 8,000 rpm for 3 min, washed twice, and resuspended in M9 medium to adjust at OD_600_ of 0.2. These bacterial suspensions were diluted 1:300 into control group, SCF (50, 50, 3 μg/mL SCF for CR-PA, MDR-PA, and S-PA, respectively) with and without glutamine (20 mM) groups. For calcium effect, calcium was added to a final concentration of 0, 4, 30, 40, and 50 μg/mL in each group. For pH effect, pH was adjusted into pH 6.0, pH 7.0, pH 8.0, and 9.0 in each group. pH was adjusted using the following buffers: homopiperazine-N-bis-2-(ethanesulfonic acid) (HOMOPIPES) for pH 5.0, 2-(N-morpholino) ethanesulfonic acid (MES) for pH 6.0 and 7.0, 3-(N-morpholino) propanesulfonic acid (MOPS) for pH 8.0, and 3-[(1,1-dimethyl-2-hydroxyethyl)amino]-2-hydroxypropanesulfonic acid (AMPSO) for pH 9.0 (Research Organics or Sigma). Cultures were incubated at 37°C for 6 h. Note that pH was maintained within ± 0.1 units in pH-related experiment. Surviving bacteria were quantified by plating. Each sample was analyzed in quadruplicate.

### 2.11 Effect of bacterial density on killing efficacy

The effect of bacterial density on killing efficacy was carried out as described previously (CLSI, 2023). Nineteen isolates (15 CR-PA and 4 MDR-PA) and ATCC27853 were used in this experiment. Bacteria were cultured as the same as above. Bacterial densities of 10^3^, 10^4^, 10^5^, 10^6^, and 10^7^ CFU/mL were prepared using M9 medium for each strain and divided into control group, SCF (50, 50, 3 μg/mL SCF for CR-PA, MDR-PA, and S-PA, respectively) with and without glutamine (20 mM) groups. Cultures were incubated at 37°C for 6 h, and surviving bacteria were quantified by plating. Each condition was analyzed in quadruplicate.

### 2.12 Determination of protein binding rate of SCF using equilibrium dialysis

The determination was carried out as described previously (Singhvi et al., 1977). The dialysis cup equipped with a dialysis membrane was inspected to confirm the absence of leakage, disinfected by wiping with alcohol, and air-dried. Subsequently, Human serum containing 20 μg/mL SCF was added to the dialysis cup, followed by the addition of glutamine to achieve final concentrations of 2.5, 5, 10, and 20 mM, respectively, resulting in a total volume of 500 μL. The lid was tightly sealed, and the dialysis cup was immersed in 5 mL of PBS buffer, ensuring complete submersion of the dialysis membrane at the bottom. The system was dialyzed at 4°C for 72 h. The SCF concentration in the serum was quantified using the Oxford cup assay. The protein binding rate of SCF was calculated using the following formula: Protein binding rate (%) = (Total SCF in serum − 5 × 2 × SCF concentration in buffer)/(total SCF in serum) × 100%.

### 2.13 Post-antibiotic effect

Post-antibiotic effect (PAE) was carried out as described previously (Li et al., 2023). The PAE assay was designed to assess the sustained inhibitory effect of bacteria after exposure to antibiotics using an experimental method based on time-kill curve studies. Single colonies from plates were picked and incubated overnight in 50 mL LB conical flasks at 37°C, 200 rpm on a constant temperature shaker. The transferred bacteria were grown to 1.5 × 10^8^ CFU/mL (OD_600_ of 0.2). Bacteria were collected and washed three times in saline. Using MHB to resuspend the bacterial bodies, 500 μL of the above bacterial solution (7.5 × 10^7^) was added to 4.5 mL of M9 medium, and the M9 tubes contained the antibiotics to be assayed at concentrations of 1/2× MIC, 1× MIC, 2× MIC, and 4× MIC with and without 20 mM glutamine. These bacteria were incubated at 37°C for 1 h. After incubation, 100 μL of the bacterial solution was diluted 1,000-fold, and then 100 μL was added to a 4.9 mL MHB tube. The diluted bacterial solution was placed in a 37°C incubator to continue incubation, and sampling was performed at 0, 1, 2, 4, 6, 8, and 12 h. Dilution spot plates were counted. The PAE was calculated by taking time as the horizontal coordinate and log10 (number of bacteria) CFU/mL as the vertical coordinate, fitting the growth curve with a regression curve, and taking the regression equation with the *R*^2^ closest to 1. The PAE was calculated as PAE = *T* − *C*, where *T* is the time required to increase the number of bacteria by 10-fold after the removal of SCF or SCF + glutamine; *C* is the time required for the increase of the number of bacteria by 10-fold after the control bacteria diluted by 1,000-fold. It was determined whether exogenous glutamine prolonged the PAE of SCF by comparing PAE of SCF with PAE of SCF + glutamine.

### 2.14 Delayed resistance development

The assay was carried out as described previously (Zhao et al., 2021). Three or four colonies of *P. aeruginosa* standard strain (ATCC27853), MDR-PA0566 and CR-PA0506 were picked from the LB plate and then inoculated into 5 mL LB tubes at 37°C, 200 rpm overnight for 16 h to saturation. The saturated bacteria were collected by centrifugation at 8,000 rpm for 3 min and washing with 0.85% saline for three times and then added M9 medium to adjust the OD_600_ to 0.2. These bacteria were divided into black control group, 1/2 MIC SCF group and 1/2 MIC SCF + 20 mM glutamine. These bacteria were incubated at 200 rpm at 37°C for 6 h, followed by centrifugation to remove the supernatant and incubation in fresh antibiotic-free LB medium for 12 h. The passaging steps were repeated for 15 cycles. The MIC was determined at each cycle.

### 2.15 Resistance frequency assay

This assay was carried out as described previously (Li et al., 2023). MDR-PA0562, CR-PA0506 and ATCC 27853 were used for resistance frequency assay experiment. The strains were cultured as described above. Bacterial solution with OD_600_ = 0.2 was diluted into 1:100 using M9 medium. Then 200 μL was coated in the LB plates containing 2× MIC–16× MIC concentrations with and without 20 mM glutamine. The plates were incubated at 37°C for 16 h and drug-resistant mutant colonies were counted. Mutation frequency = number of resistant colonies/total viable cells in inoculum. The impact of glutamine on SCF-induced mutagenesis was assessed as: Fold decrease in SCF-induced mutations by glutamine = (Glutamine + SCF mutation rate)/(SCF-only mutation rate).

### 2.16 Animal studies

This assay was carried out as described previously (Zhao et al., 2021). Animal experiments were conducted using 6–8-week-old BALB/c mice (20 ± 1 g). These animals were fed for 1 week and then infected with 1 × 10^8^ CFU of CR-PA 0576 or 2 × 10^8^ CFU of CR-PA 1252. One hour later, these infected mice were divided into saline control group, SCF group, meropenem group, and SCF + glutamine group, 10 for each group. The groups were separately intravenously injected with saline solution, 100 mg/kg meropenem, 400 mg/kg SCF, or 400 mg/kg SCF + 600 mg/kg glutamine. These mice were monitored for 7 days. Protocols were approved by the Institutional Animal Care and Use Committee. Mice were acclimatized for 1 week, and conformed to institutional animal care and use policies. Mouse experiments were conducted in strict accordance with the recommendations outlined in the Guide for the Care and Use of Laboratory Animals by the National Institutes of Health. All protocols were reviewed and approved by the Institutional Animal Care and Use Committee (IACUC) of Sun Yat-sen University (Approval No. SYSU-IACUC-2020-B126716).

### 2.17 Statistical analysis

All statistical analyses were performed using GraphPad Prism v7. Data were tested for normality, and pairwise comparisons were conducted using a two-tailed Student’s *t*-test unless otherwise specified. Error bars represent mean ± standard deviation (SD). Differences were considered at two levels of *p* < 0.05 and *p* < 0.01.

## 3 Results

### 3.1 Antimicrobial susceptibility testing of antibiotics

**Figure 1:**
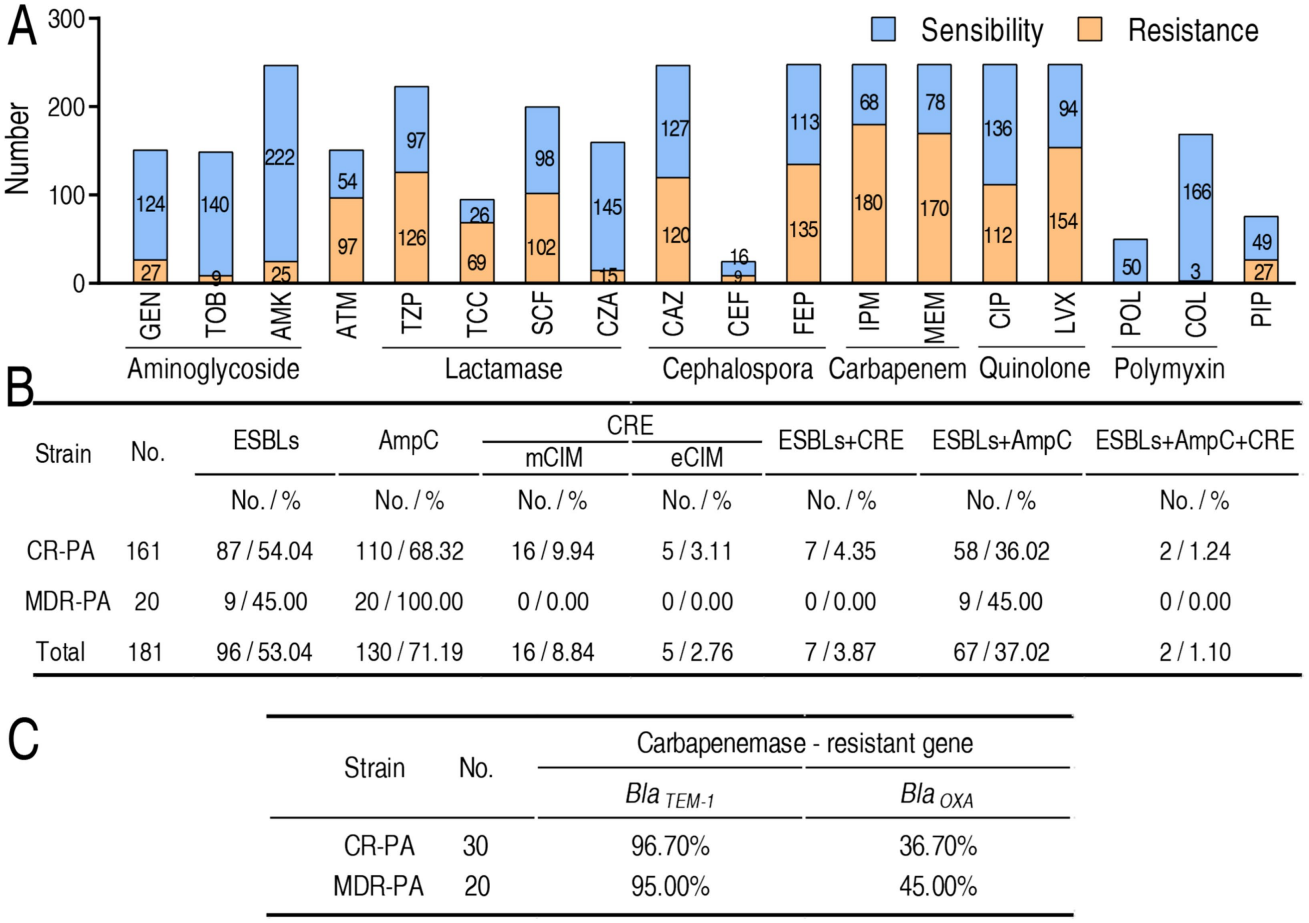
MIC and frequency of β-lactamases and their genes in *P. aeruginosa* isolates tested. **(A)** Number of drug sensitivity or resistance-based MIC value according to CLSI breakpoints. Intermediate is included in resistance. Specifically, the number of intermediate as following: gentamicin (GEN), 3 (11.99%); tobramycin (TOB), 0 (0.00%); amikacin (AMK), 10 (4.05%); aztreonam (ATM), 22 (14.57%); piperacillin tazobactam (TZP), 40 (17.94%); ticarcillin clavulanic acid (TCC), 13 (13.68%); cefoperazone sulbactam (SCF), 47 (23.5%); ceftazidime avibactam (CZA), 0 (0.00%); ceftazidime (CAZ), 33 (13.36%); cefoperazone (CEF), 3 (12.00%); cefepime (FEP), 69 (27.82%); imipenem (IPM), 5 (2.02%); meropenem (MEM), 17 (6.85%); ciprofloxacin (CIP), 43 (17.34%); levofloxacin (LVX), 42 (16.94%); polymyxin (POL), 0 (0.00%); colistin (COL), 0 (0.00%); piperacillin (PIP), 7 (9.21%). **(B)** Frequency of carbapenemase (CRE), ESBLs, and AmpC in 161 CR-PA and 20 MDR-PA. **(C)** Frequency of genes encoding CRE, ESBLs, and AmpC in 30 CR-PA and 20 MDR-PA.

Antimicrobial susceptibility testing was conducted across 259 *P. aeruginosa* clinical isolates obtained from four third-class hospitals in China (Supplementary Table S1). Minimum inhibitory concentration (MIC) values were determined in accordance with Clinical and Laboratory Standards Institute (CLSI) guidelines (CLSI, 2023), categorizing isolates as either susceptible or resistant (including intermediate resistance). Based on MIC profiles for eight antibiotics spanning seven classes (Figure 1), strains were categorized into three groups: (1) antibiotic-sensitive *P. aeruginosa* (S-PA), (2) multidrug-resistant but carbapenem-sensitive *P. aeruginosa* (MDR-PA), and (3) multidrug-resistant carbapenem-resistant *P. aeruginosa* (CR-PA). Classification criteria were defined as follows: S-PA: Susceptibility to all tested antimicrobial agents, MDR-PA: Resistance to ≥3 antimicrobial classes, and CR-PA: Resistance to ≥3 antimicrobial classes plus meropenem and/or imipenem. Predominant resistance phenotypes included nonsusceptibility to gentamicin, tobramycin, amikacin, aztreonam, piperacillin-tazobactam, ticarcillin-clavulanic acid, cefoperazone-sulbactam, cefotaxime-avibactam, ceftazidime, cefoperazone, cefepime, imipenem, meropenem, ciprofloxacin, levofloxacin, polymyxin B, colistin, and piperacillin (Figure 1A).

### 3.2 Resistance phenotypes of β-lactamases and their genotypes

β-lactamase profiling was performed across 20 MDR-PA and 161 CR-PA clinical isolates, focusing on three enzyme classes: (1) plasmid-encoded extended-spectrum β-lactamases (ESBLs) mediating extended-spectrum cephalosporin hydrolysis, (2) AmpC cephalosporinases conferring broad-spectrum cephalosporin resistance (Bush and Bradford, 2020), and (3) carbapenemases enabling carbapenem degradation. Carbapenemase detection employed modified carbapenem inactivation methods (mCIM for general carbapenemases; eCIM specifically identifying metallo-β-lactamases) (Lasko et al., 2020). Among the 20 MDR-PA, 9 (45.00%), 20 (100.00%), 0 (0.00%) exhibited ELSBs, AmpC, and carbapenemases, respectively, while out of the 161 CR-PA, 87 (54.04%), 110 (68.32%), 16 (9.94%), and 5 (3.11%) displayed ELSBs, AmpC, and mCIM- and eCIM-carbapenemases, respectively. In addition, double positivity was 9 (45.00%, ESBLs + AmpC for MDR-PA), 7 (4.35%, ESBLs + carbapenemases for CR-PA), 58 (36.02%, ESBLs + AmpC for CR-PA), and triple positivity was 2 (1.24%, ESBLs + AmpC + carbapenemases for CR-PA) (Figure 1B). Furthermore, *Bla_TEM-1_* and *Bla_OXA-1_* were selected for amplification in 30 CR-PA and 20 MDR-PA. The TEM-1 acts by hydrolysing the β-lactam ring of penicillins, cephalosporins and related antibiotics (Salverda et al., 2010). OXA-1 counts for most of the resistance to carbapenems (Walther-Rasmussen and Høiby, 2006). Totally, 19 (95.00%) *Bla*_TEM-1_ and 9 (45.00%) *Bla*_TEM-1_ were positive in 20 MDR-PA and 29 (96.70%) *Bla*_TEM-1_ and 11 (36.70%) *Bla*_OXA-1_ were positive in 30 CR-PA (Figure 1C).

### 3.3 The combined therapeutic efficacy of SCF with glutamine and determination of KE50 and KE90 values across the clinical isolates

**Figure 2:**
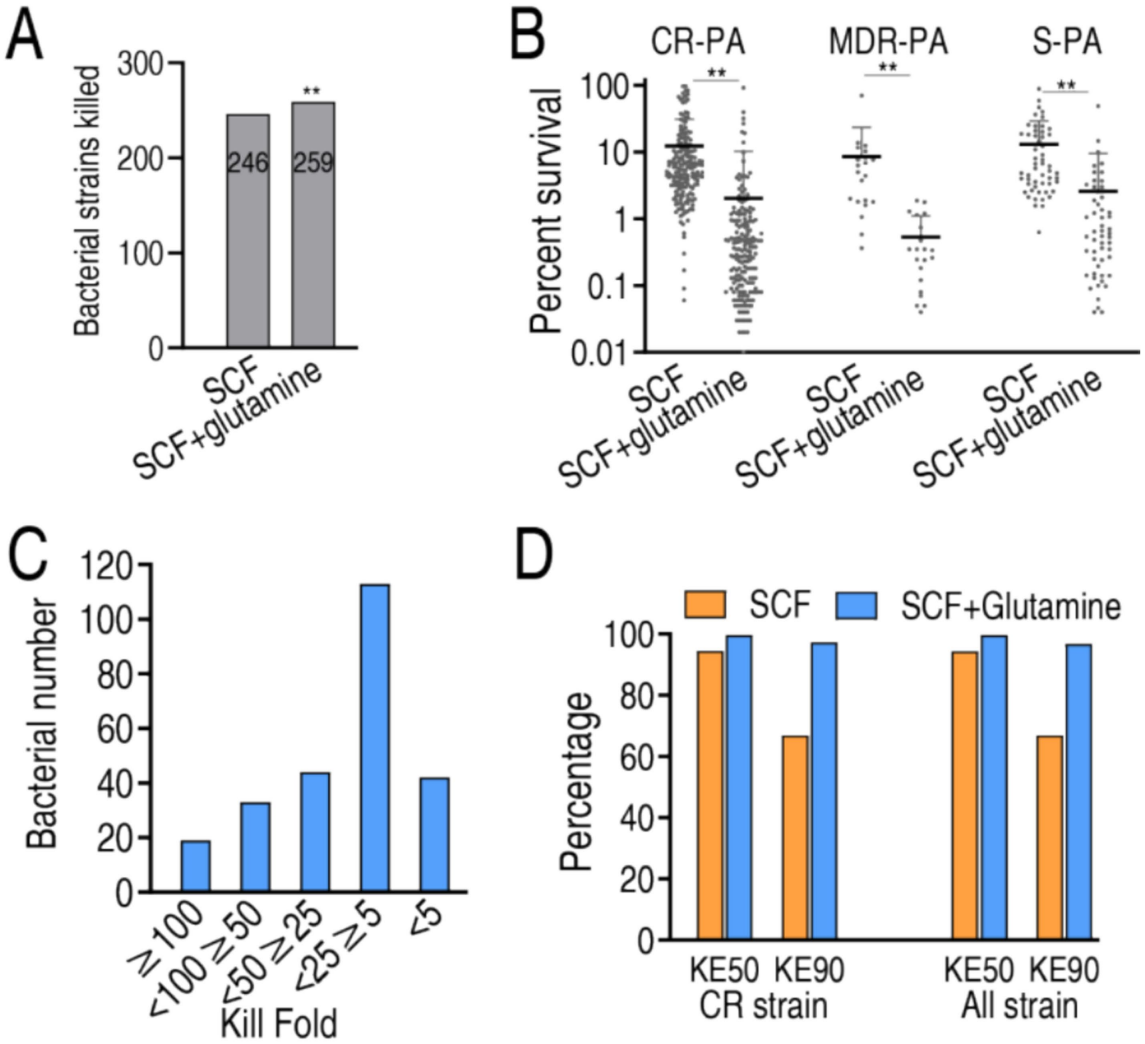
SCF killing in the absence or presence of glutamine. **(A)** Number of bacterial strains killed in SCF treatment with or without glutamine. **(B)** Percent survival of 185 CR-PA, 20 MDR-PA, and 54 S-PA isolates in the presence of 50 μg/mL, 50 μg/mL, 3 μg/mL SCF, respectively, with or without glutamine. **(C)** Number of the indicated killing folds based on data **(A)**. **(D)** KE_50_ and KE_90_ in data **(A)**. KE_50_ and KE_90_ represent the percentage of bacteria killed at the 50 and 90% points among the isolated strains, respectively. Data are presented as mean ± standard error of the mean (SEM). ^**^p < 0.01.

To evaluate the bactericidal enhancement of SCF by glutamine against *P. aeruginosa* clinical isolates, bacterial survival was analyzed in 259 strains, across three experimental cohorts: 54 antibiotic-sensitive (S-PA), 20 multidrug-resistant (MDR-PA), and 185 carbapenem-resistant (CR-PA) strains. The study design included untreated control (no SCF/glutamine), SCF monotherapy and SCF-glutamine combination therapy. Both treatment arms demonstrated significant bactericidal activity relative to controls across all strain types. Among the 259 bacterial strains, SCF alone eliminated 246 (95%), but the combination of SCF and glutamine achieved complete eradication (100%) (Figure 2A). Comparative analysis between SCF monotherapy and combination therapy revealed three distinct interaction profiles: synergistic efficacy (superior bactericidal effect versus SCF alone), additive efficacy (equivalent activity to SCF monotherapy) and indifferent interaction (reduced activity compared to SCF alone). Among the 259 clinical isolates, the combination therapy exhibited synergistic efficacy in 253 strains (97.7%), additive effects in four strains (1.5%) and indifferent responses in two strains (0.8%) (Figure 2B). Synergistic isolates were further categorized based on the fold-enhancement ratio of bactericidal efficacy (combination therapy vs. SCF monotherapy) into five tiers: 44 strains (<5-fold enhancement), 12 strains (5–25-fold), 45 strains (25–50-fold), 31 strains (50–100-fold), 21 strains (>100-fold) (Figure 2C).

MIC_50_ and MIC_90_ values, critical pharmacodynamic parameters for antimicrobial susceptibility reporting in multi-isolate studies (Lee et al., 2023), were not applicable in this experimental system due to the use of non-growth-permissive M9 medium. Instead, bactericidal efficacy was quantified through kill efficacy indices (KE_50_ and KE_90_), defined as the percentage of bacteria killed at the 50 and 90% points among the isolated strains, respectively. The tested 259 isolates exhibited 94.51 and 99.65% KE50 and 72.50 and 96.85% KE90 in SCF alone and the synergy, respectively. Among the 185 CR-PA, KE50 was 94.37 and 99.67% and KE90 was 74.67 and 97.48% in SCF without and with glutamine, respectively (Figure 2D). This pharmacodynamic profile confirms the superior bactericidal efficacy of SCF-glutamine combination therapy compared to SCF monotherapy.

### 3.4 Time-kill kinetic profiling with glutamine supplementation

**Figure 3:**
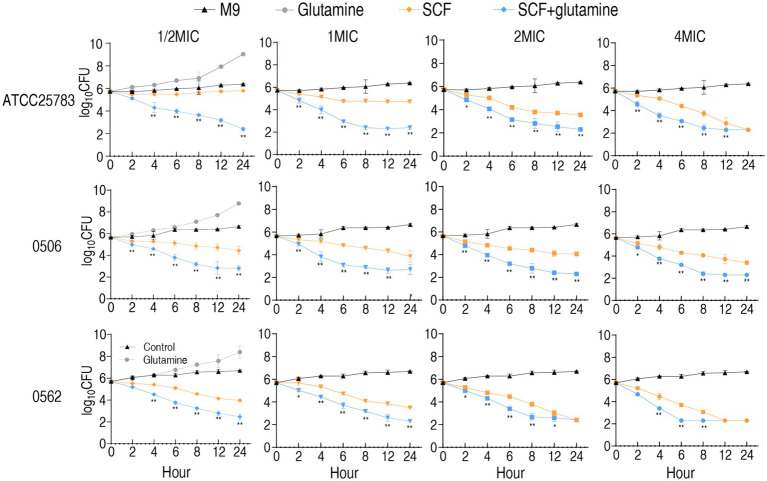
Time kill-kinetics of ATCC25783, CR-PA 0506, and MDR-PA 0562 in the presence of 1/2 MIC, 1 MIC, 2 MIC, and 4 MIC of SCF with or without 20 mM glutamine for the indicated incubation time. MIC of ATCC25783, 0506, and 0562 to SCF were 4, 32, and 16 μg/mL, respectively. Since the SCF bactericidal assays for the four different MIC doses of each strain were performed simultaneously, they shared the same blank control group. To avoid redundancy, the blank control and glutamine-only data are only listed under the 1/2 MIC section. Please refer to it accordingly. Data are presented as mean ± standard error of the mean (SEM). ^*^p < 0.05 and ^**^p < 0.01 compared to SCF group.

To delineate the bactericidal kinetics of SCF against *P. aeruginosa*, time-kill assays were conducted using reference strain ATCC 25783 (antibiotic-sensitive), clinically isolated MDR-PA 0562 and CR-PA 0506. Test concentrations included 0.5×, 1×, 2×, and 4× MIC of SCF, with parallel evaluation of glutamine’s adjuvant effects. Bactericidal activity exhibited time-dependent progression across all concentrations in both treatment arms, with two exceptions of against ATCC 25783 at subinhibitory concentrations (1/2× MIC) and 1× MIC. Comparative analysis revealed significantly enhanced killing kinetics in glutamine-supplemented versus unsupplemented media across all tested SCF concentrations when only glutamine without SCF increased bacterial growth (Figure 3). These findings demonstrate that glutamine potentiates SCF-mediated eradication of *P. aeruginosa* strains (S-PA, MDR-PA, CR-PA) through time-dependent pharmacodynamic enhancement.

### 3.5 *In vivo* efficacy of glutamine-enhanced SCF therapy

**Figure 4:**
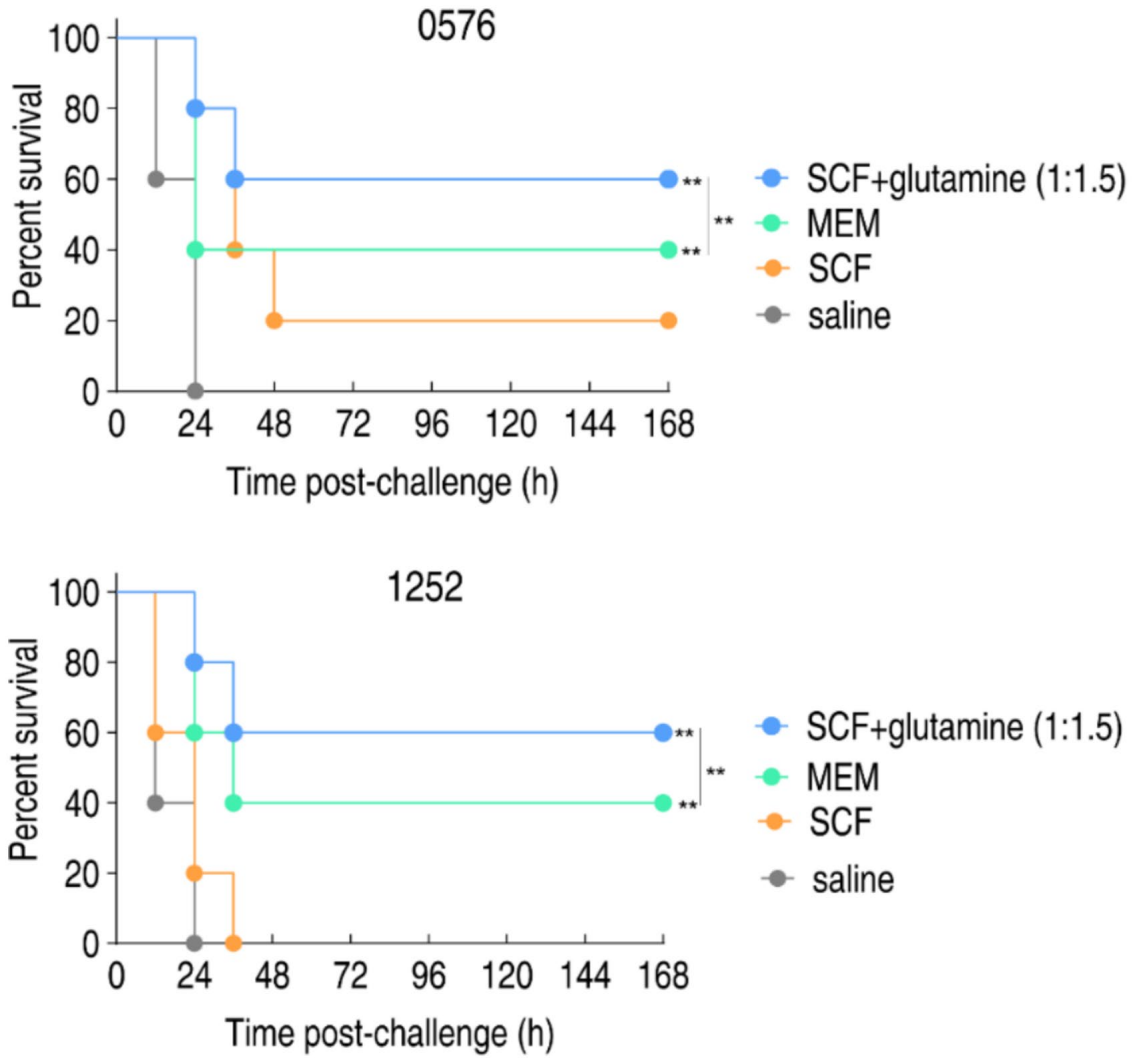
Percent survival of mice infected with CR-PA 0576 or CR-PA 1252 in the absence or presence of SCF, glutamine or/and both. Mice were intraperitonally infected with 1 × 10^8^ CFU of CR-PA 0576 or 2 × 10^8^ CFU of CR-PA 1252 and divided into four groups, respectively. The groups were separately intravenously injected with saline solution, 100 mg/kg meropenem (MEM), 400 mg/kg SCF, or 400 mg/kg SCF + 600 mg/kg glutamine (1:1.5) in an hour later. ^**^p < 0.01.

To evaluate the therapeutic potential of glutamine-potentiated SCF *in vivo*, murine infection models were established using carbapenem-resistant *P. aeruginosa* strains CR-PA0576 and CR-PA1252. Infected mice (*n* = 10 per group) were treated with saline control, meropenem monotherapy, SCF monotherapy and SCF-glutamine combination. Treatments were administered intravenously commencing one-hour post-infection, with survival monitored over 7 days. For CR-PA0576 infection, SCF-glutamine, meropenem and SCF alone achieved 60% survival, 40% survival, and 20% survival, respectively. For CR-PA1252 infection, SCF-glutamine, meropenem and SCF alone achieved 60% survival, 40% survival and 0% survival, respectively (Figure 4). This survival profile demonstrates significant *in vivo* therapeutic enhancement of SCF by glutamine against carbapenem-resistant *P. aeruginosa*.

### 3.6 Glutamine enhances cefoperazone pharmacokinetics to overcome efflux and hydrolysis

**Figure 5:**
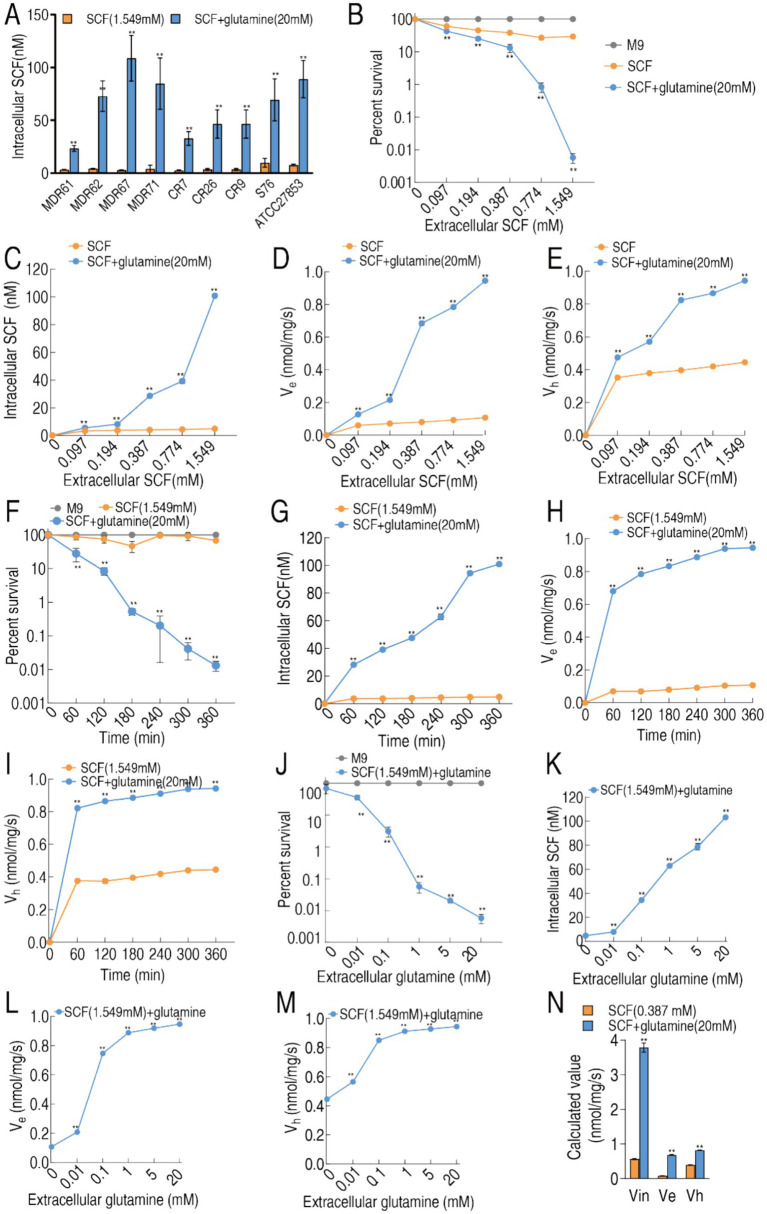
Pharmacokinetics of cefoperazone in MDR-PA 0562 in the presence or absence of 20 mM glutamine. **(A)** Intracellular SCF concentration of these indicated bacterial strains in the presence of 1.594 mM extracellular SCF with or without 20 mM glutamine. **(B–E)** Relationship of increasing extracellular SCF concentrations with percent survival **(B)**, influx **(C)**, efflux **(D)**, and hydrolysis **(E)** in the presence or absence of 20 mM glutamine. **(F–I)** Relationship of increasing incubation periods with percent survival **(F)**, influx **(G)**, efflux **(H)**, and hydrolysis **(I)** in the presence or absence of 20 mM glutamine plus SCF (1.549 mM). **(J–M)** Relationship of extracellular glutamine concentrations with percent survival **(J)**, influx **(K)**, efflux **(L)**, and hydrolysis **(M)** in the presence SCF (1.549 mM). **(N)** Calculated values (nmol/mg per second) of *V*_in_, *V*_e_, and *V*_h_ in MDR-PA 0562 at *C*_0_ = 1× MIC. Data are means ± SEM from three biological replicates. ^**^p < 0.01 compared to SCF group. **(J–M)** ^**^p < 0.01 compared to 0 (without glutamine).

To investigate glutamine-mediated potentiation of SCF uptake, intracellular drug concentrations were quantified in nine clinically representative strains: 4 MDR-PA, 3 CR-PA, and 2 S-PA. Glutamine supplementation significantly increased intracellular SCF accumulation across all tested strains (Figure 5A). A pharmacokinetic model using MDR-PA 0562 (AmpC-producing) revealed dynamic drug disposition as follows: bacterial viability inversely correlated with extracellular SCF concentration (Figure 5B). Cefoperazone accumulation positively correlated with extracellular concentration, efflux pump activity and enzymatic hydrolysis increased proportionally, glutamine-enhanced regimens demonstrated superior drug retention (Figures 5C–E). At 1.549 mM SCF, viability was reduced, and cefoperazone uptake, efflux, and hydrolysis were increased in a time-dependent manner in the presence of glutamine, but kept stable or weak change in the absence of glutamine (Figures 5F–I). Dose-response analysis using fixed SCF (1.549 mM) with graded glutamine (0–10 mM) over 6 h demonstrated intracellular cefoperazone, and cefoperazone efflux and hydrolysis were increased with increasing glutamine (Figures 5J–M). When extracellular cefoperazone concentration is equal to bacterial MIC, the intracellular cefoperazone concentration is defined as zero. The relationship of enzymatic hydrolysis (*V*_h_), influx (*V*_in_), and efflux (*V*_e_) is designated as *V*_in_ = *V*_h_ + *V*_e_ (Kojima and Nikaido, 2013). Therefore, the equation of *V*_in_ ≈ 5.56 was obtained, when exogenous glutamine increased *V*_in_ from 0.46 to 3.78 nmol/mg per second, *V*_h_ from 0.38 to 0.47 nmol/mg per second, *V*_e_ from 0.071 to 0.21 nmol/mg per second (Figure 5N).

### 3.7 Glutamine-mediated metabolic reprogramming profiling

**Figure 6:**
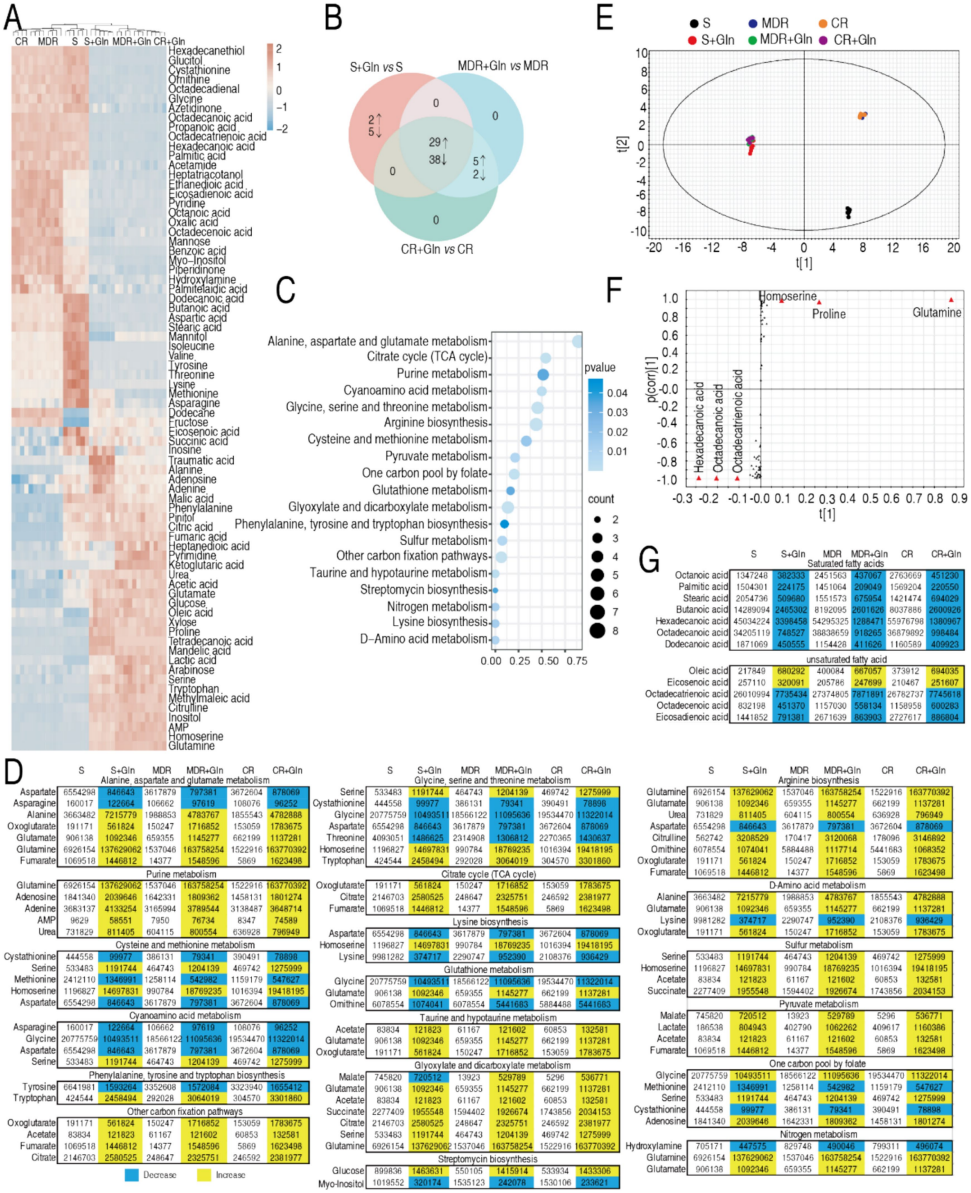
Metabolomics of S-PA, MDR-PA, and CR-PA in the absence or presence of glutamine and regulation of unsaturated/saturated fatty acids. **(A)** Heat map showing differential abundance of metabolites (row) using R language. Yellow and blue indicate the increase and decrease of the metabolites relative to the mean and SD of the row metabolite level, respectively (see color scale). **(B)** Venn diagram for comparison of differential metabolites of S-PA, MDR-PA, and CR-PA with and without glutamine (Gln). **(C)** Pathway enrichment of overlapped differential metabolites among S-PA, MDR-PA, and CR-PA with glutamine. **(D)** Integrative analysis of metabolites in significantly enriched pathways. Yellow and blue colors indicate increased and decreased metabolites (*p* < 0.05), respectively. **(E)** OPLS-DA of metabolomes in S-PA, MDR-PA, and CR-PA with and without glutamine (Gln). Each dot represents the biological and technical replicate analysis of samples in the plot. **(F)** S-plot generated from OPLSDA. Predictive component *p* [1] and correlation *p* (corr) [1] differentiate S-PA, MDR-PA, and CR-PA with glutamine (Gln) from S-PA, MDR-PA, and CR-PA without glutamine. **(G)** Saturated and unsaturated fatty acids identified by GC-MS in data **(A)**.

To elucidate the metabolic basis of glutamine-potentiated bactericidal activity, GC-MS-based metabolomic analysis was conducted across three *P. aeruginosa* strains: reference strain ATCC 27853, CR-PA 0506, and MDR-PA 0562. The experimental design included treatment groups (± glutamine supplementation) and replicates (quadruplicate biological replicates with duplicate technical replicates per strain). Technical reproducibility was validated by inter-replicate correlation coefficients (*R*^2^ = 0.996; Supplementary Figure S1A), confirming robust experimental reproducibility. Global metabolomic profiling identified 91 metabolites, stratified into functional categories via KEGG pathway classification: 22% amino acids, 13% carbohydrate, 6% nucleotide, 35% lipid, and 24% others (Supplementary Figures S1B,C). Comparative analysis revealed significant metabolic rewiring, with 74 metabolites (81.3%) exhibiting differential abundance profiles between glutamine-supplemented and control conditions (Figure 6A). Unsupervised clustering analysis stratified the strains into two distinct metabolic clusters based on glutamine supplementation states. Within the two groups, the biological replicates of ATCC27853 were grouped and the biological replicates of CR-PA 0506 and MDR-0562 were combined. Note that the distance between ATCC27853 with and without glutamine is the smallest between the two groups (Figure 6A). *Z*-score analysis was implemented to quantify deviations of the 74 differentially expressed metabolites between individual measurements and the mean values in three *P. aeruginosa* strains: the standard strain ATCC 25783 (S-PA), multidrug-resistant strain MDR-PA 0562, and carbapenem-resistant strain CR-PA 0506, under both glutamine-supplemented and glutamine-depleted conditions (Supplementary Figure S2). Comparative analysis revealed distinct regulation patterns: ATCC 25783 exhibited 31 upregulated and 43 downregulated metabolites, while both MDR-PA 0562 and CR-PA 0506 showed 34 upregulated and 40 downregulated metabolites when comparing glutamine-treated versus untreated conditions. A three-set Venn diagram analysis demonstrated metabolite regulation overlap across strains. Core conserved responses included 29 consistently upregulated and 38 persistently downregulated metabolites shared among all three strains. Strain-specific patterns emerged with five upregulated and two downregulated metabolites uniquely co-occurring in the two resistant strains (MDR-PA 0562 and CR-PA 0506), while the standard strain ATCC 25783 displayed two uniquely upregulated and five uniquely downregulated metabolites (Figure 6B). Pathway enrichment analysis demonstrated that the 67 overlapped metabolites were functionally associated with 19 distinct metabolic pathways (Figure 6C). Strikingly, glutamine-induced metabolic reprogramming effectively restored nearly all downregulated metabolites in drug-resistant strains (MDR-PA 0562 and CR-PA 0506) to baseline levels comparable to or exceeding those in the drug-sensitive ATCC 25783 strain (Figure 6D). Pattern recognition was employed to identify biomarkers through orthogonal partial least squares discriminant analysis (OPLS-DA). The OPLS-DA model classified *t*[1] into two distinct subgroups: glutamine-supplemented and non-supplemented, while *t*[2] clustered with ATCC 27853 (sensitive strain) and segregated from resistant strains MDR-PA 0562 and CR-PA 0506 (Figure 6E). An S-plot identified discriminating variables using cutoff values of ≤0.05 for absolute covariance *p* and ≥0.5 for correlation *p* (corr). For *t*[1], downregulated hexadecanoic acid, octadecatrienoic acid, and octadecenoic acid were identified as biomarkers, alongside upregulated glutamine, homoserine, and proline (Figure 6E). Conversely, *t*[2] analysis revealed downregulated methionine and upregulated adenosine monophosphate (AMP), citric acid, and phenylalanine as biomarkers (Figures 6E,F). Since a substantial proportion of the identified biomarkers were fatty acid metabolites in *t*[1], we systematically compiled a comprehensive list of all detected fatty acid metabolites (Figure 6G). Analytical profiling demonstrated that glutamine supplementation triggered selective downregulation of all saturated fatty acids (SFAs), while unsaturated fatty acids (UFAs) showed differential regulation patterns comprising both upregulation and downregulation clusters (Figure 6G).

### 3.8 The role of UFA/SFA ratio in regulating membrane permeability for drug uptake

**Figure 7:**
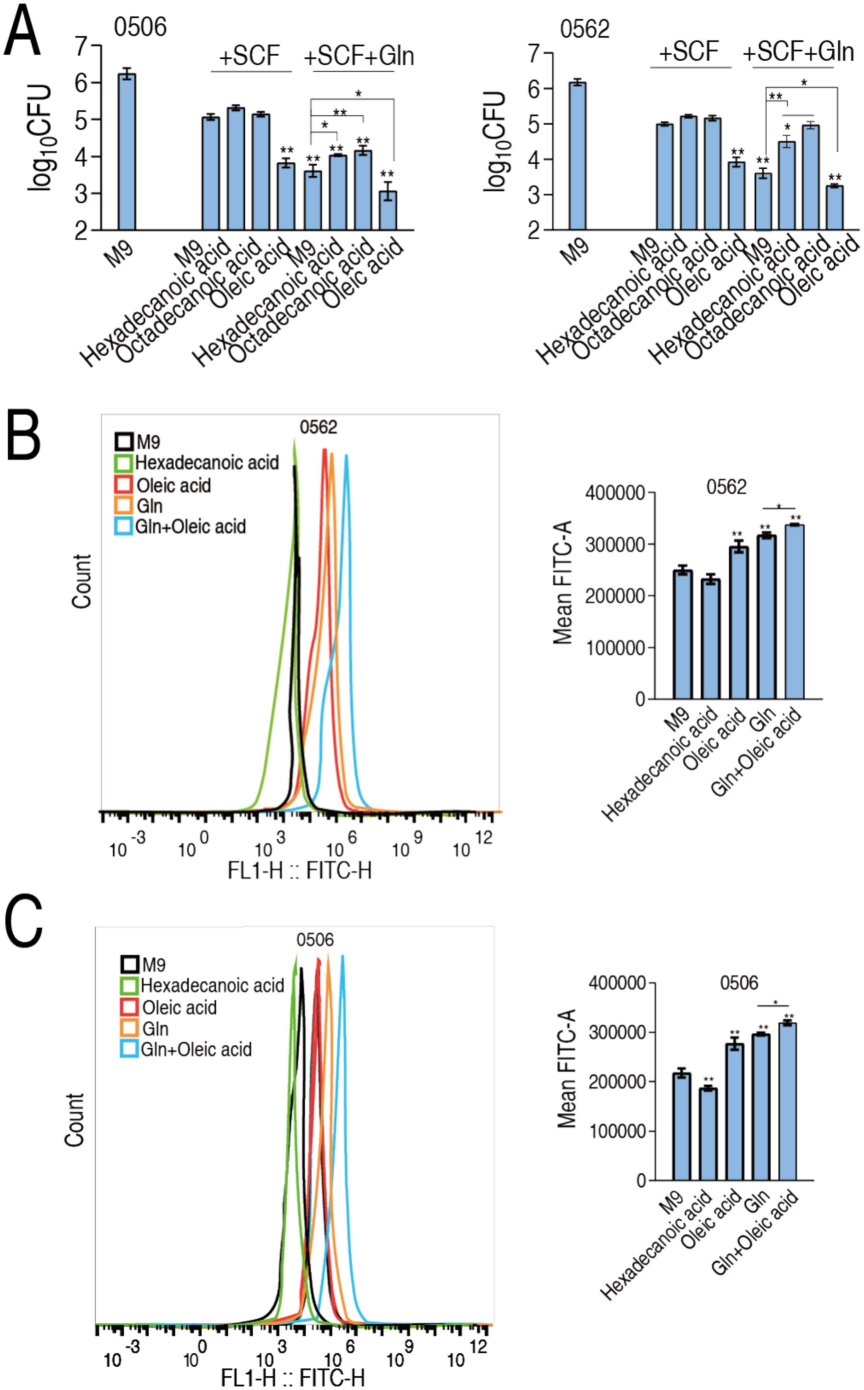
Modulation of UFA/SFA ratio to membrane permeability and drug uptake. **(A)** Number of CR-PA0506 and MDR-PA 0562 in the absence or presence of SCF or/and glutamine (Gln) or the indicated metabolites. **(B,C)** Membrane permeability of MDR-PA 0562 (I) and CR-PA0506 (J) in the absence or presence of the indicated metabolites. Data are presented as mean ± SEM. ^*^p < 0.05 and ^**^p < 0.01 compared to SCF group.

This regulatory dichotomy suggests glutamine’s bactericidal potentiation mechanism in SCF may involve strategic modulation of membrane biophysics through an elevated UFA/SFA ratio, thereby increasing membrane fluidity and permeability to enhance SCF uptake. To functionally validate this membrane remodeling hypothesis, we conducted fatty acid supplementation experiments using two SFAs (hexadecanoic acid and octadecanoic acid) and one UFA (oleic acid) in the absence or presence of glutamine plus SCF. Notably, SFAs failed to enhance SCF-mediated bacterial eradication in the absence of glutamine and paradoxically inhibited glutamine’s bactericidal enhancement in the presence of glutamine. Conversely, oleic acid potentiated SCF efficacy (Figure 7A). The experimental data establish that glutamine-mediated elevation of the unsaturated-to-saturated fatty acid (UFA/SFA) ratio constitutes a critical mechanistic driver of SCF bactericidal potentiation, likely through enhanced membrane permeability facilitating compound uptake. To validate this, membrane permeability was measured in MDR-PA0562 and CR-PA0506 in the presence of glutamine, hexadecanoic acid, oleic acid, or glutamine + oleic acid. Compared to glutamine—which increased membrane permeability—hexadecanoic acid reduced it, while oleic acid had no effect. In contrast, the combination of glutamine and oleic acid slightly elevated membrane permeability (Figures 7B,C). These results suggest that glutamine enhances membrane permeability by increasing the UFA/SFA ratio.

### 3.9 Glutamine delays post-antibiotic effect of SCF and plasma protein binding with SCF

**Figure 8:**
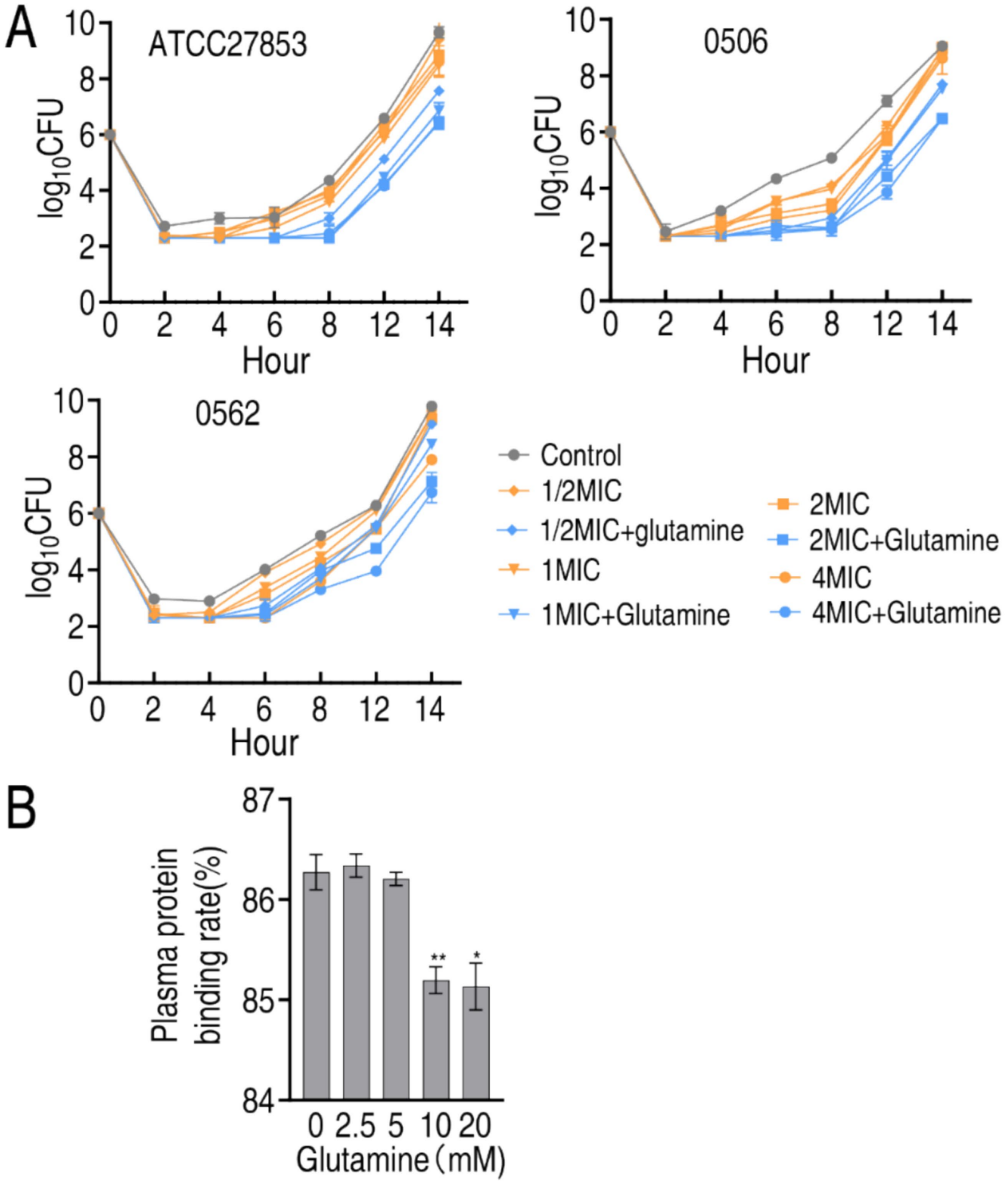
Effect of glutamine on PAE, MIC, and plasma protein binding of SCF. **(A)** PAE of SCF to ATCC27853, CR-PA 0506, and MDR-PA 0562 in the absence of presence of 20 mM glutamine plus the indicated MIC of SCF and incubation time. MIC of ATCC27853, CR-PA 0506, and MDR-PA 0562 to SCF were 4, 32 and 16 μg/mL, respectively. There were differences in viability at *p* < 0.01 between SCF group and SCF + glutamine group. **(B)** Plasma protein binding rate in the absence or presence of glutamine. Data are presented as mean ± SEM. ^**^p < 0.01, ^*^p < 0.05.

PAE as one characteristic of pharmacodynamics means the delayed regrowth of bacteria following exposure to an antibiotic. To test whether glutamine has the role, post-antibiotic effect experiment was performed against ATCC27853, CR-AP 0506, and MDR-AP 0566. After bacteria were exposed to 1/2 MIC, 1 MIC, 2 MIC or 4 MIC of SCF, differential growth was observed. Specifically, the difference in the growth was significant from 6 h and persisted until 14 h in the three strains (Figure 8A). On the other hand, human plasma proteins were isolated to test whether glutamine influences the binding of these proteins with SCF. The plasma protein binding was 86.27% in the absence of glutamine. However, the binding was reduced to 85.13% when 10 and 20 mM glutamine was used (Figure 8B). These results indicate that glutamine not only delays PAE of SCF, but also reduces the plasma protein binding, suggesting the mechanisms by which glutamine potentiates SCF killing.

### 3.10 Effect of environmental factors on glutamine-potentiated SCF killing

Calcium and pH as environmental factors were selected to test whether they affect the glutamine-potentiated killing. A total of 20 strains were used in the two assays including 15 CR-PA (0503, 0506, 0509, 0511, 0513, 0518, 0521, 0538, 0539, 0540, 0541, 0546, 0548, 0549, 0550), 4 MDR-PA (0560, 0562, 0563 and 0566), and 1 S-PA (ATCC27853). These strains were divided into blank control without SCF and glutamine, SCF group, and SCF + glutamine group. For calcium assay, because higher than 50 μg/mL calcium concentrations in M9 medium cause precipitation, 0 μg/mL, 4 μg/mL, 30 μg/mL, 40 μg/mL, 50 μg/mL of calcium were used. Viability of these strains was lower in SCF + glutamine group than SCF group and SCF group than black control in each of 0–50 calcium concentrations (Supplementary Figure S3A). For pH assay, the killing efficacy was performed in pH 5, pH 6, pH 7, pH 8, and pH 9. In every pH, lower survival of these strains was measured in SCF + glutamine than SCF, which exhibited lower survival than blank control (Supplementary Figure S3B). These results indicate that calcium and pH do not affect the glutamine-potentiated killing.

### 3.11 Effect of bacterial amount on glutamine-potentiated SCF killing

To test whether bacterial amount influences the glutamine-potentiated killing, cultures of the above 20 strains were diluted into 10^7^, 10^6^, 10^5^, 10^4^, and 10^3^ CFU/mL and used for viability measurement in blank control, SCF group, and SCF + glutamine group. The viability was reduced with decreasing bacterial amount. Particularly, no viability of the 20 strains with 10^3^, and 10^4^ CFU/mL was detected in for both SCF group and SCF + glutamine group; however, lower viability of the 20 strains (except for 0506 and 0549 in 10^7^ CFU/mL, at which similar survival was detected) was measured in SCF + glutamine group than SCF group when 10^7^, 10^6^, and 10^5^ CFU/mL cultures were used (Supplementary Figure S4). These results indicate that the glutamine-potentiated killing is related to bacteria number.

### 3.12 Glutamine postposes SCF resistance

**Figure 9:**
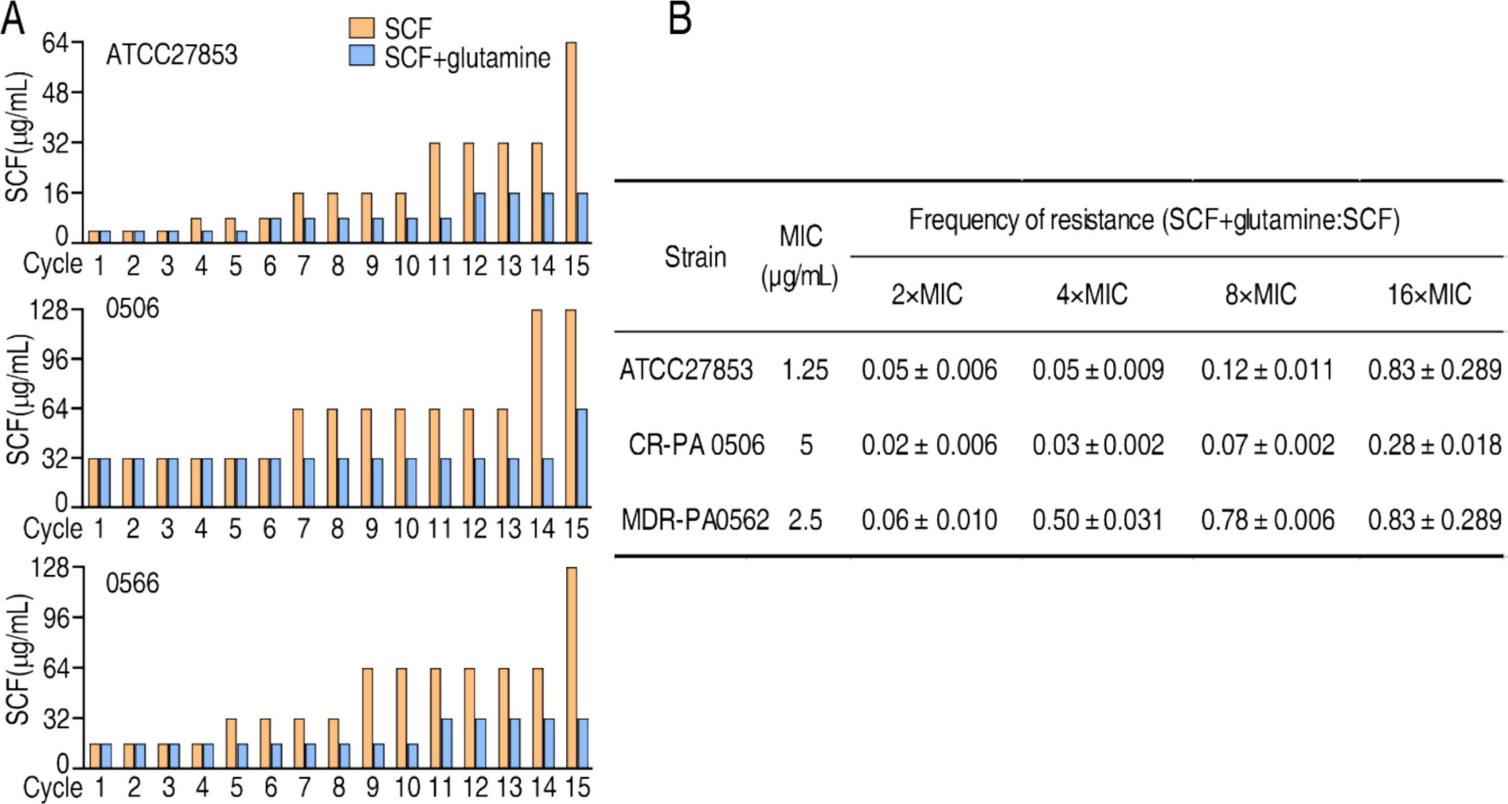
Effect of glutamine on MIC of SCF and mutation. **(A)** MIC of cycled ATCC27853, CR-PA 0506, and MDR-PA 0566 in the absence or presence of glutamine, SCF, or/and both. Starting MIC: 4 μg/mL for ATCC700603; 16 μg/mL for 0566; 32 μg/mL for 0506. Bacteria were cultured in M9 medium with or without 1/2 MIC of SCF for 15 cycles. MIC was measured each cycle. **(B)** Bacterial mutation frequency in the absence or presence of glutamine.

Bacterial antibiotic resistance is an inevitable consequence of exposure to antibiotics. To know whether glutamine postposes SCF resistance, ATCC27853, CR-PA0506, and MDR-PA0566 were passaged in medium with 1/2 SCF in the absence or presence of glutamine. A total of 15 cycles were carried out. MIC was easured in every cycle. MIC of the three strains was increased as the cycles go up in medium with and without glutamine. However, higher MIC was measured in the absence than presence of glutamine, being 64 MIC vs. 16 MIC, 128 MIC vs. 64 MIC, 128 MIC vs. 32 MIC of ATCC27853, CR-PA0506, and MDR-PA0566, respectively (Figure 9A). Therefore, glutamine postposes the resistance to SCF.

### 3.13 Determination of bacterial mutation frequency in the absence or presence of glutamine

To better understand the role of glutamine in the inhibition of SCF resistance, mutation frequency of ATCC27853, CR-PA0506 and MDR-PA0562 is investigated. SCF resistance increased in the bacterium with a low mutation frequency after exposure to SCF alone. However, the resistance and mutation frequency were reduced after exposure to the combination of SCF and glutamine. Specifically, the mutation frequency ratio of SCF + glutamine vs. SCF alone was 0.05 ± 0.006, 0.05 ± 0.009, 0.12 ± 0.01, and 0.83 ± 0.2800 for ATCC27853; 0.02 ± 0.006; 0.03 ± 0.002, 0.07 ± 0.002, and 0.28 ± 0.018 for CR-PA0506; 0.06 ± 0.010, 0.60 ± 0.010, 0.78 ± 0.006, and 0.83 ± 0.2800 for MDR-PA0562, respectively, in 2 MIC, 4 MIC, 8 MIC, 16 MIC, 32 MIC, 64 MIC (Figure 9B). These results indicate that glutamine reduces the mutation frequency of *P. aeruginosa* exposed to SCF.

## 4 Discussion

Enhancing the killing efficacy of existing antibiotics is a highly feasible strategy to combat antibiotic-resistant bacteria, particularly given the stagnation in new antibiotic discovery (Peng et al., 2023, 2015b; Brinkmann et al., 2022; Li S. H. et al., 2025). Recent advances in metabolic reprogramming of resistant bacteria—reversing antibiotic-resistant metabolic states into antibiotic-sensitive ones by reprogramming metabolites—have shown promise in restoring susceptibility to existing antibiotics (Jiang et al., 2023; Chen et al., 2023; Cheng et al., 2019; Kuang et al., 2021; Su et al., 2021). Building on the finding that glutamine enhances ampicillin-mediated killing of multidrug-resistant (MDR) *E. coli* (Zhao et al., 2021), this study investigates the preclinical pharmacodynamics of sulbactam/cefoperazone (SCF) combined with glutamine against *P. aeruginosa* (PA), including MDR-PA and CR-PA strains. Glutamine significantly potentiates SCF activity, achieving higher bactericidal efficacy against S-PA, MDR-PA, and CR-PA strains compared to SCF alone. The killing effect intensifies with increased SCF doses, prolonged incubation times, and reduced bacterial colony-forming units (CFU), while remaining unaffected by variations in calcium concentrations or pH. Despite the production of extended-spectrum β-lactamases (ESBLs), AmpC, and carbapenemases in some PA strains, the SCF-glutamine combination outperforms SCF monotherapy. Therefore, the synergistic use highlights the way in metabolite-enabled killing of MDR-PA and CR-PA by existing antibiotics. Importantly, the combination of glutamine and SCF makes full use of two advantages: (1) promotes drug uptake by increasing membrane permeability, likely through metabolic reprogramming that elevates the unsaturated-to-saturated fatty acid ratio (Li H. et al., 2025), a factor linked to antibiotic efficacy (Su et al., 2021; Li S. H. et al., 2025; Ye et al., 2024; Xue et al., 2024). (2) Sulbactam inhibits β-lactamase-mediated degradation, preserving cefoperazone activity. Together, these effects synergistically boost intracellular cefoperazone concentrations, enhancing bacterial killing. To our knowledge, whether glutamine affects membrane permeability was unknown before.

Bacterial susceptibility to antibiotics occurs when intracellular drug concentrations surpass the lethal threshold (Peng et al., 2023). Consequently, the dynamics of intracellular drug concentrations with versus without glutamine elucidate the mechanism by which glutamine potentiates SCF to effectively eradicate antibiotic-resistant pathogens. Glutamine enhances drug uptake in a dose-dependent manner. Although concurrent increases in efflux pump activity and enzymatic drug degradation are observed, the augmented drug influx outweighs these counteractive mechanisms, resulting in a net elevation of intracellular drug concentrations. Once this concentration exceeds the lethal threshold, bacterial death ensues. The bactericidal effect correlates directly with intracellular antibiotic levels, demonstrating a concentration-dependent killing efficacy. These findings not only rationalize the glutamine-mediated enhancement of SCF activity against MDR-PA and CR-PA but also underscore that augmenting drug influx represents a viable strategy to overcome resistance mechanisms, even in pathogens with heightened efflux or hydrolytic enzyme expression (e.g., β-lactamases). Notably, bacteria harboring extended-spectrum β-lactamases (ESBLs), AmpC, or carbapenemases—such as carbapenem-resistant *Acinetobacter baumannii*, *Pseudomonas aeruginosa*, and *Enterobacteriaceae* and third-generation cephalosporin-resistant *Enterobacteriaceae*—are classified as critical-priority pathogens by the WHO due to their global threat (Antimicrobial Resistance Collaborators, 2022; Guo et al., 2022). The integration of antimicrobial agents with metabolites to potentiate drug uptake is emerging as a next-generation therapeutic strategy to combat these multidrug-resistant superbugs, circumventing the limitations of traditional antibiotic development pipelines (Peng et al., 2023).

Beyond potentiating intracellular cefoperazone concentrations, glutamine prolongs the post-antibiotic effect (PAE) of SCF and reduces its plasma protein binding. PAE, a critical pharmacodynamic parameter for determining antibiotic dosing regimens (Mohamed et al., 2021), is increasingly integrated into the design of optimized antimicrobial therapies (Yan et al., 2022). Although cefoperazone exhibits time-dependent bactericidal activity (Tilanus and Drusano, 2023), glutamine extends the PAE of SCF, suggesting that glutamine-mediated PAE prolongation may allow for extended dosing intervals while maintaining therapeutic efficacy. Additionally, glutamine reduces plasma protein binding of SCF, a factor critical for optimizing drug exposure, as only the unbound fraction exerts pharmacological activity (Ewoldt et al., 2023). Reduced binding liberates more free SCF, enhancing its bactericidal action. At concentrations of 10–20 mM, glutamine facilitates SCF release from plasma proteins, thereby increasing the bioactive fraction. These dual mechanisms—prolonged PAE and enhanced free drug availability—position the SCF-glutamine combination as a promising next-generation therapeutic strategy for combating multidrug-resistant *P. aeruginosa* (MDR-PA) and carbapenem-resistant *P. aeruginosa* (CR-PA).

Bactericidal susceptibility testing to evaluate the combined effect of SCF and glutamine was conducted via plate count assays in M9 minimal medium rather than minimum inhibitory concentration (MIC) testing in nutrient-rich LB medium. This approach avoids confounding factors arising from endogenous glutamine and other nutrients in LB medium, which are metabolized by bacteria and could obscure the specific contribution of exogenous glutamine. Notably, glucose was omitted from the M9 medium to eliminate its known antibiotic-potentiating effects (Allison et al., 2011). While plate count assays are widely employed to assess antibiotic killing efficacy, they do not yield traditional pharmacodynamic parameters such as MIC50/MIC90 (the drug concentrations inhibiting 50% or 90% of isolates, respectively). However, the killing efficacy indices KE50 and KE90 can be derived from the plate count data, representing the minimum bactericidal efficiency (expressed as a percentage) required to kill 50 and 90% of the tested bacterial strains, respectively. These metrics provide a quantitative framework to evaluate bactericidal activity, complementing MIC-based assessments.

Glutamine reduces gene mutation frequency, a phenomenon that can be attributed to the role of bacterial metabolism in antibiotic resistance and susceptibility (Zhao et al., 2021). There are complex interactions between antibiotic resistance, metabolism, and gene mutation. The most common canonical mechanisms of antibiotic resistance, including target modification, enzymatic inactivation, and reduced antibiotic uptake, may also exert indirect metabolic effects. A typical example is the downregulation of cell membrane porins not only decreases antibiotic influx but also limits nutrient uptake, thereby altering metabolic activity. Metabolism contributes to antibiotic resistance through differential metabolic gene expression, activation of alternative pathways, and insertion of metabolic genes (Ahmad et al., 2025). For instance, the extensive diversity of metabolic gene mutations observed in over 3,500 clinical *Escherichia coli* isolates highlights the broad metabolic flexibility that facilitates evolutionary adaptation (Lopatkin et al., 2021). Additionally, activation of the SOS response can introduce mutations, including those in antibiotic targets that confer resistance (Ahmad et al., 2025). Conversely, exogenous glutamine has been shown to suppress mutations in genes involved in glutamine and purine metabolism (Zhao et al., 2021), suggesting a metabolic checkpoint that constrains mutagenesis.

## 5 Conclusion

The preclinical pharmacodynamics of SCF with glutamine against *P. aeruginosa*, especially MDR-PA and CR-PA is demonstrated through high-throughput killing assays in M9 medium, time-kill curves, and analysis of calcium/pH effects and bacterial numbers. It is documented that the SCF-glutamine combination exhibited significantly enhanced killing *in vitro* and *in vivo* compared to SCF alone. *In vitro* testing of 259 clinical isolates revealed synergistic effects in 253 strains (97.7%), including 13 strains resistant to SCF monotherapy. *In vivo* evaluation using two CR-AP strains showed a 40–60% increase in survival rates, surpassing the efficacy of meropenem. Furthermore, this study investigates mechanistic insights through analyses of intracellular drug dynamics (uptake and retention), post-antibiotic effect (PAE) modulation, plasma protein binding interactions, and metabolic state alterations. The results demonstrate that enhanced drug influx surpasses counteractive efflux and enzymatic degradation—a phenomenon directly attributed to glutamine’s ability through reprograming antibiotic-resistant metabolic states into antibiotic-sensitive ones to elevating the ratio of unsaturated/saturated fatty acids for increasing membrane permeability. These findings position the SCF-glutamine combination as a promising therapeutic candidate against MDR-PA and CR-PA, offering a dual strategy to overcome resistance: enhancing intracellular drug accumulation and mitigating resistance mechanisms. This approach aligns with the growing emphasis on metabolite-antibiotic synergism as a next-generation solution for antimicrobial-resistant pathogens.

## Data Availability

The original contributions presented in the study are included in the article/Supplementary material, further inquiries can be directed to the corresponding authors.
