# Supplementary material for: Glutamine potentiates cefoperazone-sulbactam activity against Pseudomonas aeruginosa by increasing membrane permeability and cellular uptake
Source: Front Microbiol. 2025 Jul 3;16:1631646. doi: 10.3389/fmicb.2025.1631646 (PMC12267172; doi:10.3389/fmicb.2025.1631646)
Supplement: Supplementary file 1 [file Data_Sheet_1.docx]

**Supplementary Table and Figures**

**Table S1 Bacterial isolates used in this study**

| Hospital | No. | S-PA | | MDR-PA | | CR-PA | |
| --- | --- | --- | --- | --- | --- | --- | --- |
|  |  | No. | % | No. | % | No. | % |
| Xiamen University Affiliated Zhongshan Hospital, Xiamen, China | 76 | 6 | 7.89 | 16 | 21.05 | 53 | 71.05 |
| Hanzhong Central Hospital, Hanzhou, China | 48 | 18 | 37.5 | 1 | 2.08 | 29 | 60.42 |
| The First People's Hospital of Foshan City, Foshan, China | 110 | 18 | 16.36 | 3 | 2.73 | 89 | 80.91 |
| Third Affiliated Hospital of Sun Yat sen University, Gunagzhou, China | 25 | 12 | 48 | 0 | 0 | 13 | 52 |
| Total | 259 | 54 | 20.85 | 20 | 7.72 | 185 | 71.43 |

No. Number

S-PA, antibiotic-sensitive *P. aeruginosa;* MDR-PA, multidrug-resistant *P. aeruginosa;* CR-PA, carbapenem-resistant *P. aeruginosa.*


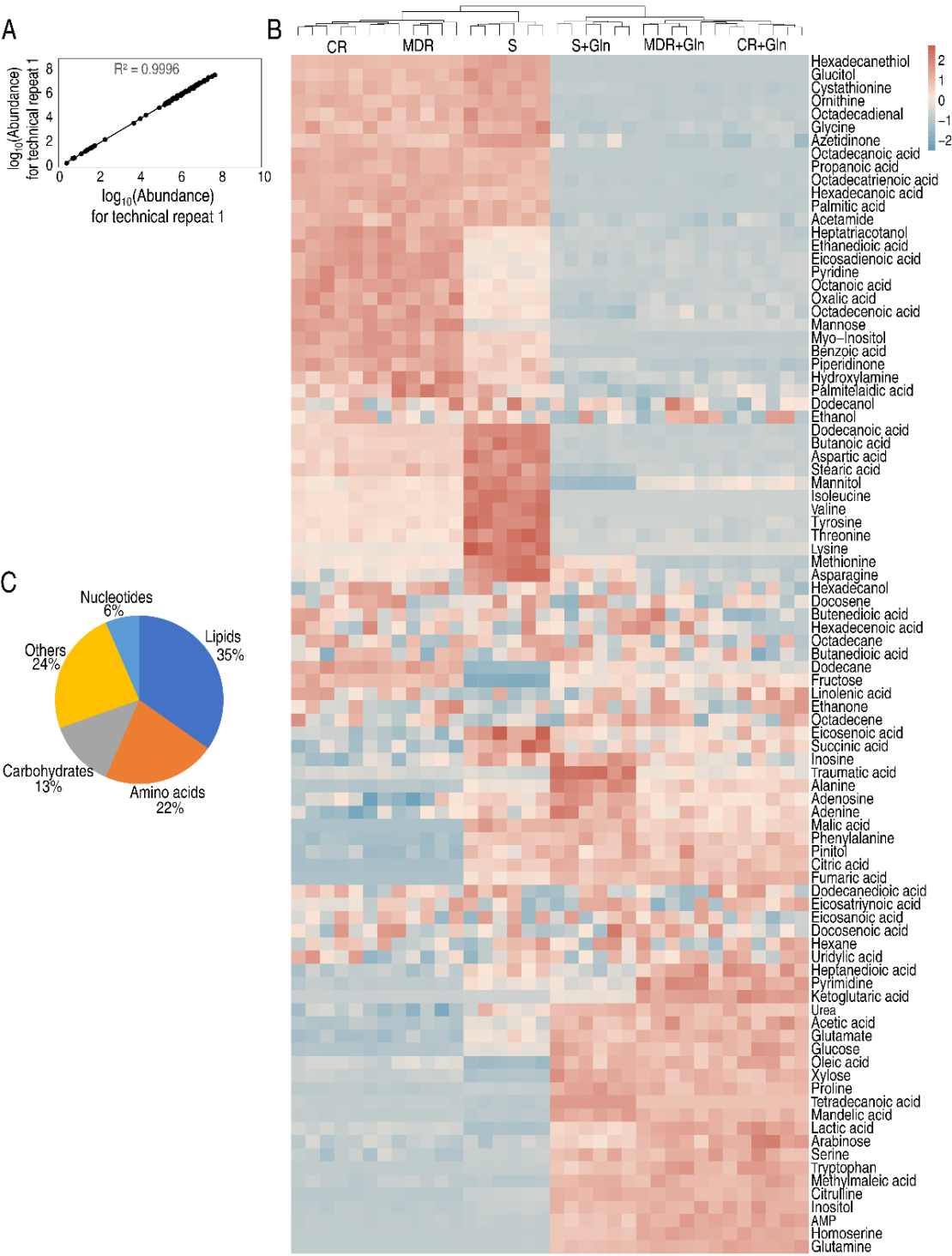


**Figure S1 Metabolic profiles in S-PA, MDR-PA, and CR-PA with and without glutamine**. A, Correlation coefficient between technical replicates. B, Heat map of unsupervised hierarchical clustering of all metabolites (row). Yellow and blue colors indicate increase and decrease of the metabolites scaled to mean and standard deviation of row metabolite level, respectively (see color scale). C, Categories of all identified metabolites


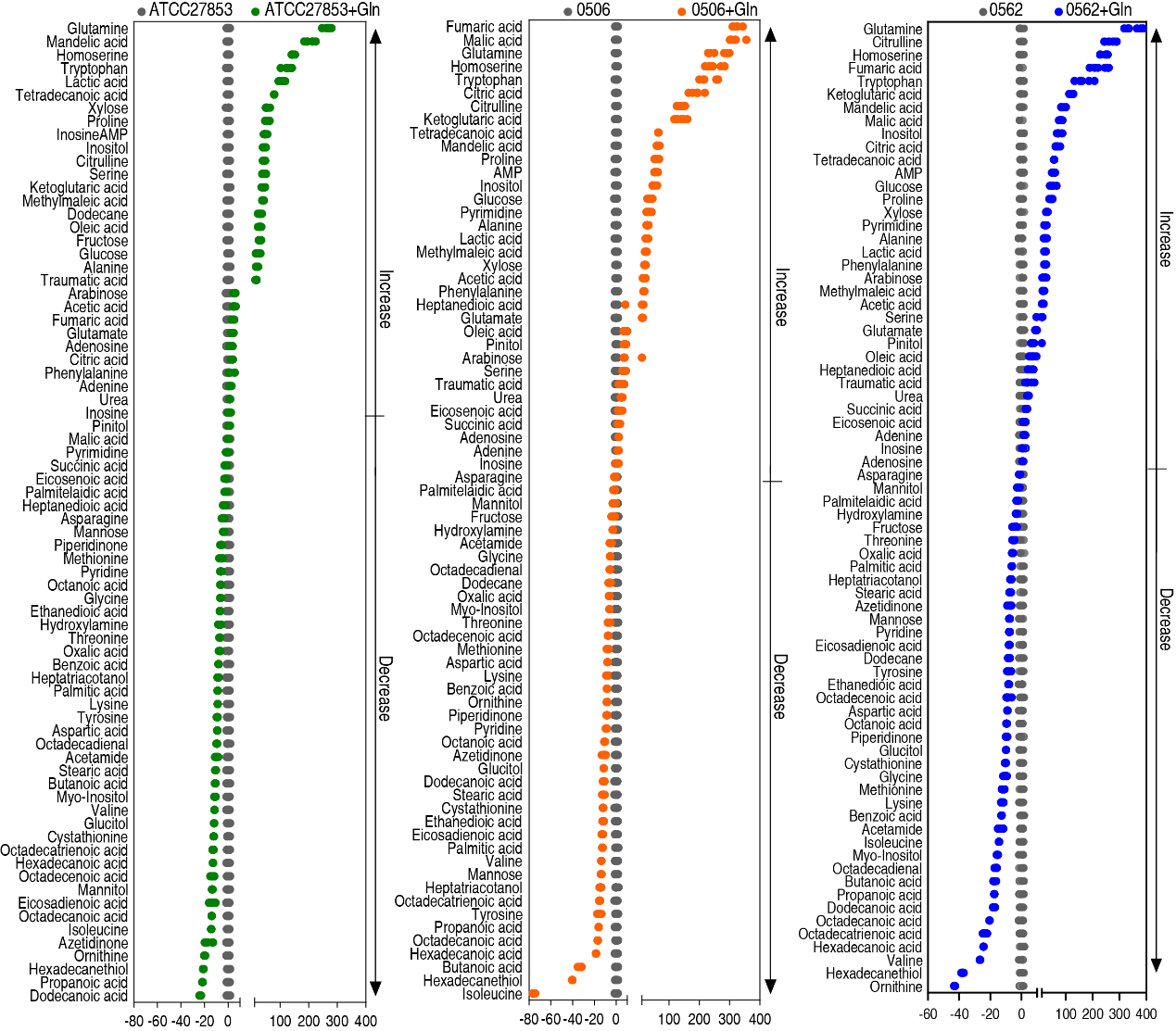


**Figure S2 Z-value plot of differential metabolites**


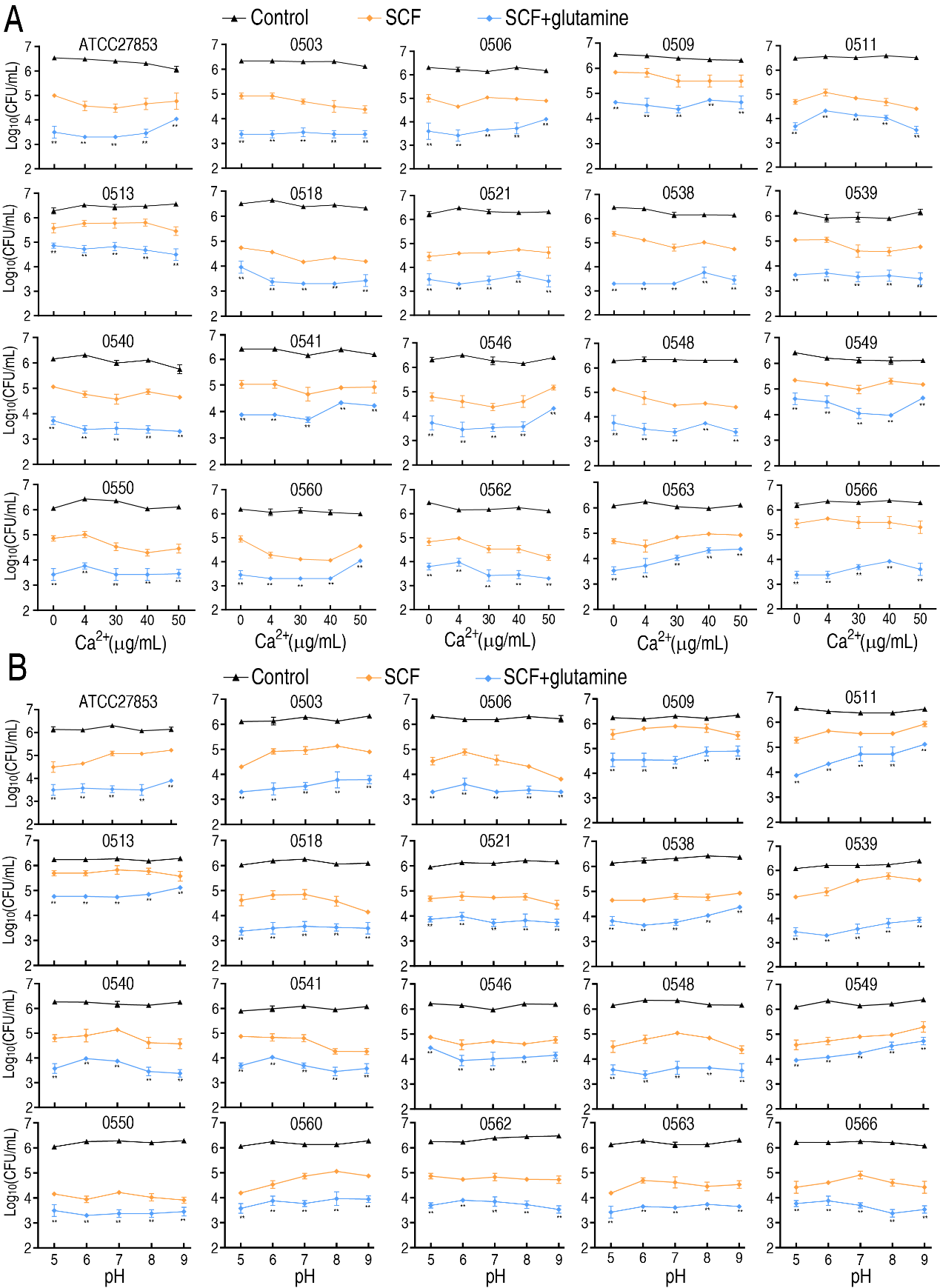


**Figure S3. Effect of calcium and pH on percent survival of ATCC27853 and 19 isolates in medium with the indicated concentrations of calcium (A) or the indicated pH (B) plus 20 mM glutamine. 50 μg/mL, 50 μg/mL, 3 μg/mL SCF for** **CR-PA (0503, 0506, 0509, 0511, 0513, 0518, 0521, 0538, 0539, 0540, 0541, 0546, 0548, 0549, 0550), MDR-PA (0560, 0562, 0563 and 0566), and ATCC27853, respectively.**

Data are presented as mean ± SEM. ** P < 0.01 compared to SCF group.


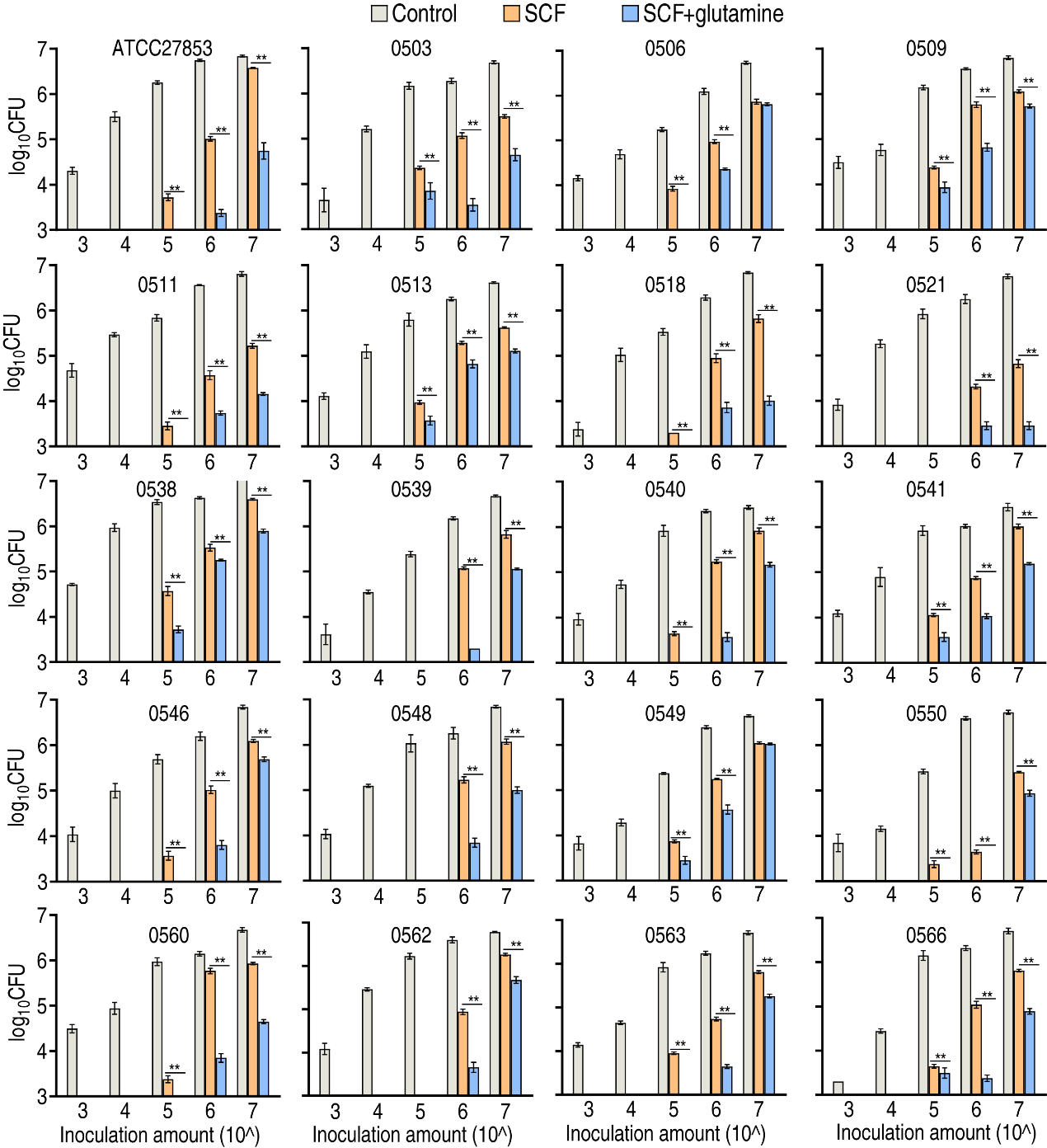


**Figure S4. Percent survival of ATCC27858 and 19 isolates with the indicated bacterial CUF/mL in the absence or presence of 20 mM glutamine and 50 μg/mL, 50 μg/mL, 3 μg/mL SCF for** **CR-PA (0503, 0506, 0509, 0511, 0513, 0518, 0521, 0538, 0539, 0540, 0541, 0546, 0548, 0549, 0550), MDR-PA (0560, 0562, 0563 and 0566), and ATCC27853, respectively.**

Data are presented as mean ± SEM. ** P < 0.01.
